# Single‐Cell Transcriptome Identifies Drug‐Resistance Signature and Immunosuppressive Microenvironment in Metastatic Small Cell Lung Cancer

**DOI:** 10.1002/ggn2.202100060

**Published:** 2022-03-04

**Authors:** Jing Zhang, Haiping Zhang, Lele Zhang, Dianke Li, Mengfan Qi, Liping Zhang, Huansha Yu, Di Wang, Gening Jiang, Xujun Wang, Xianmin Zhu, Peng Zhang

**Affiliations:** ^1^ Department of Thoracic Surgery Shanghai Pulmonary Hospital, School of Medicine Tongji University Shanghai 200433 China; ^2^ Department of Medical Oncology Shanghai Pulmonary Hospital School of Medicine Tongji University Shanghai 200433 China; ^3^ Central Laboratory Shanghai Pulmonary Hospital School of Medicine Tongji University Shanghai 200433 China; ^4^ Department of Pathology Shanghai Pulmonary Hospital School of Medicine Tongji University Shanghai 200433 China; ^5^ SJTU‐Yale Joint Center for Biostatistics School of Life Science and Biotechnology Shanghai Jiao Tong University Shanghai 200240 China; ^6^ Shanghai Institute for Advanced Immunochemical Studies ShanghaiTech University Shanghai 201210 China

**Keywords:** heterogeneity, natural killer cells, single‐cell RNA sequencing, small cell lung cancer, tumor microenvironments

## Abstract

Small cell lung cancer (SCLC) is a deadly neuroendocrine malignancy with high metastasis. However, the heterogeneity of metastatic SCLC at the single‐cell level remains elusive. The single‐cell transcriptome of a total of 24 081 cells in metastatic lymph node samples from seven SCLC patients via endobronchial ultrasound‐guided transbronchial needle aspiration (EBUS‐TBNA) is examined. Genomic alterations are also examined by whole exome sequencing (WES) and the immune infiltration between SCLC and non‐SCLC (NSCLC) is compared using public single‐cell RNA sequencing (scRNA‐seq) data. It is identified that malignant cells in lymph‐node metastatic SCLC have inter‐patient and intra‐tumor heterogeneity characterized by distinct *ASCL1* and *NEUROD1* expression patterns. High expression of genes such as *FZD8* in WNT pathway is associated with drug resistance in malignant cells. Compared to NSCLC, SCLC harbors a unique immunosuppressive tumor microenvironment. Malignant cells exhibit a pattern of attenuated MHC‐I antigen presentation‐related gene expression, which is associated with relatively low proportion of exhausted T cells. Natural killer (NK) cells display impaired antitumor function with high expression of *TGFBR2*. This work characterizes the inter‐patient and intra‐tumor heterogeneity of metastatic SCLC and uncovers the exhaustion signatures in NK cells, which may pave the way for novel treatments for SCLC including immune checkpoint blockade‐based immunotherapy.

## Graphical abstract

Small cell lung cancer (SCLC) is a deadly neuroendocrine malignancy with high metastasis. However, the heterogeneity of metastatic SCLC at the single‐cell level remains elusive. Current study identifies that malignant cells in SCLC have inter‐patient and intra‐tumor heterogeneity, high expression of genes such as *FZD8* in WNT pathway is associated with drug resistance, and SCLC harbors a unique immunosuppressive tumor microenvironment.

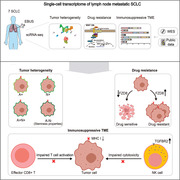

## 1 Introduction

Small cell lung cancer (SCLC) is an extremely aggressive tumor of neuroendocrine (NE) origin.^[^ ^1^ ^]^ According to the Veterans Administration Lung Study Group staging system, SCLC is categorized into limited‐stage and extensive‐stage disease based on whether it is confined to only one hemithorax.^[^ ^2^ ^]^ Based on the response to first‐line treatment, ES‐SCLC can be further classified as refractory, resistant, or sensitive SCLC. At molecular levels, genetic mutations and transcriptional dysregulation in SCLC have been characterized by combined efforts. Most SCLC tumors harbor *TP53* and *RB1* inactivation, MYC family amplification, and/or Notch pathway disruption.^[^ ^3^ ^]^ Based on the diverse expression of NE markers, SCLC tumors can be classified into NE‐high and NE‐low subtypes.^[^ ^4^ ^]^ Evidence from cell lines, primary tumors, genetically engineered mouse models, and patient‐derived xenograft (PDX) models indicates that four transcriptional regulators, that is, achaete‐scute family bHLH transcription factor 1 (*ASCL1*), neuronal differentiation 1 (*NEUROD1*), Yes1‐associated transcriptional regulator (*YAP1*) and POU class 2 homeobox 3 (*POU2F3*), can be used to further define NE‐high (*ASCL1* and *NEUROD1*) and NE‐low (*YAP1* and *POU2F3*) subtypes.^[^ ^3^, ^5^ ^]^ In addition, tumor‐propagating cell markers in SCLC, such as CD133^[^ ^6^ ^]^ and CD90,^[^ ^7^ ^]^ are associated with its aggressiveness and drug resistance. It should be noted that most of the conclusions have been drawn from analysis of bulk samples. Uncovering transcriptomic alterations at the single‐cell level will allow us to better understand the heterogeneity and inter‐cellular communication in SCLC and eventually facilitate precision medicine.

The use of platinum and etoposide chemotherapy as guided first‐line treatment has been unchanged for decades.^[^ ^8^ ^]^ Meanwhile, the 5‑year overall survival (OS) rate of ES‐SCLC remains less than 5% due to rapid progression and early metastasis.^[^ ^9^ ^]^ Unlike in non‐SCLC (NSCLC), targeted drugs against vascular endothelial growth factor (VEGF), insulin‐like growth factor 1 receptor, mechanistic target of rapamycin kinase, epidermal growth factor receptor, and hepatocyte growth factor have not achieved success in SCLC. Macrophages, T, and natural killer (NK) cells were detected in the tumor microenvironment (TME) of SCLC samples, implying their potential roles in the immunotherapy response of SCLC.^[^ ^10^ ^]^ Undoubtedly, immunotherapy becomes one of the most promising treatments for SCLC.^[^ ^11^ ^]^ The US Food and Drug Administration (FDA) recently approved anti‐PD‐L1 antibody atezolizumab for first‐line treatment with chemotherapy^[^ ^12^ ^]^ and the anti‐PD1 antibody nivolumab for third‐line treatment.^[^ ^13^ ^]^ The IMpower133 and CASPIAN studies demonstrated that immunotherapy targeting PD‐L1 significantly improved OS in patients with ES‐SCLC when used in first‐line treatment,^[^ ^12^, ^14^ ^]^ while immunotherapy targeting PD‐1 in the KEYNOTE‐604 study did not.^[^ ^11^ ^]^ This inconsistency in the results of immune checkpoint blockade‐based immunotherapy prompted us to investigate the heterogeneous cell populations, especially immune infiltrates in the TME of SCLC.

SCLC is a highly metastatic lung cancer. ES‐SCLC patients accounts for ≈70% of all SCLC patients and are diagnosed with metastasis in the common sites such as lymph nodes (LNs), brain, liver, and bones.^[^ ^15^ ^]^ The metastatic SCLC has poor responsiveness to chemotherapy and immunotherapy. Therefore, understanding the heterogeneous TME of metastatic SCLC will greatly facilitate the development of new treatments. After a comparison of different biopsy methods, we decided to investigate lymph‐node metastatic SCLC by endobronchial ultrasound‐guided transbronchial needle aspiration (EBUS‐TBNA). Compared to percutaneous lung biopsy which may cause hemorrhea and pneumothorax, EBUS‐TBNA is a safe diagnostic tool of lung cancer^[^ ^16^ ^]^ and allows the collection of samples with sufficiently high quality for downstream experiments.^[^ ^17^ ^]^ In this work, we pooled cells from EBUS‐TBNA samples collected from 7 SCLC patients. After single‐cell RNA sequencing (scRNA‐seq), we investigated the association of NE markers with inter‐ and intra‐tumoral heterogeneity, the transcriptional basis of drug resistance, and the immune infiltrates in the TME.

## 2 Results

### 2.1 Single‐Cell Transcriptomic Analysis Reveals the Heterogeneity of Metastatic SCLC in LNs

**Figure 1:**
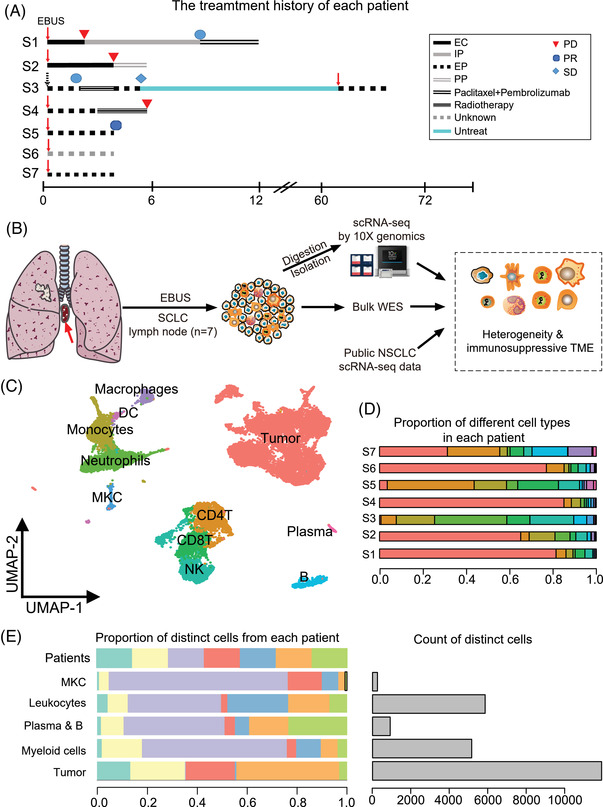
Single‐cell transcriptomic analysis reveals the heterogeneity of metastatic SCLC in the lymph node. a) Schematic illustration of the treatment received by the 7 SCLC patients, including the timepoints of EBUS biopsy for scRNA‐seq (red arrows) (EC: Etoposide+Carboplatin; IP: irinotecan+Cisplatin; EP: Etoposide+ Cisplatin; PP: Paclitaxel+ Cisplatin; PD: Progressive disease; PR: Partial response; SD: Stable disease). b) Overview of the workflow. c) UMAP visualization of different cell types isolated from 24 081 cells. d) Composition of different cell types (Tumor cells, CD4+ T cells, CD8+ T cells, natural killer (NK) cells, neutrophils, monocytes, macrophages, dendritic cells (DCs), plasma cells, B cells, and megakaryocytes (MKC) in each patient. e) Left panel shows proportions of MKCs, leukocytes, plasma and B cells, myeloid cells and malignant cells in different SCLC patients. Right panel shows numbers of each cell type (on the left panel).

We collected hilar/mediastinal lymph samples by EBUS‐TBNA from 7 SCLC patients before and during treatment (**Figure** **1**a and Table S1, Supporting Information). These patients were diagnosed with SCLC by EBUS‐TBNA sampling (Table S1, Supporting Information) and computed tomography (CT) (Figure S1, Supporting Information). Patient S1 showed the most notable drug resistance, while patient S3 had survived for more than 5 years after the first diagnosis (Figure 1a). We prepared a single‐cell suspension for scRNA‐seq using the 10X Genomics platform (Figure 1b). After quality control to exclude red blood cells, platelets, and dead cells, a total of 24 081 cells were retrieved, with an average of 2910 unique genes detected per cell. After correcting for multiple covariates with Harmony, we performed unsupervised clustering of the cells and identified 29 distinct clusters visualized by uniform manifold approximation and projection for dimension reduction (UMAP) (Figure S2a,b, Supporting Information). Using the well‐established markers of different cell lineages^[^ ^18^ ^]^ (Figure S2c, Supporting Information), we further identified different cell types, including malignant cells, T cells, B cells, NK cells, monocytes, and macrophages, in these clusters (Figure 1c and Figure S2d, Supporting Information). We found that each SCLC sample had varied percentages of distinct cell types (Figure 1d,e). However, in all samples except those from patients S3 and S5, malignant cells were the major population (32–85%), followed by leukocytes and myeloid cells (Figure 1d,e). Malignant cells showed more patient‐specific clusters than non‐malignant cells (Figure S2e,f, Supporting Information) and hence had a significantly higher Gini index (*p* = 0.018) (Figure S2g, Supporting Information). For example, the malignant cells in cluster 13 were mainly from patient S6 (Figure S2a,b,e, Supporting Information). This finding suggests that malignant cells exhibit high inter‐patient heterogeneity in SCLC which is consistent with the previous work of Stewart et al.^[^ ^19^ ^]^

As SCLC has unique genomic alterations compared to other lung cancer types,^[^ ^20^ ^]^ we then decided to investigate SCLC heterogeneity at the genomic level. We performed whole exome sequencing (WES) for all EBUS biopsies paired with the blood samples. We found that the mutational signatures of the SCLC samples were mainly determined by combination of certain mutational processes such as exposure to tobacco mutagens, DNA‐double‐strand break repair deficiency, and spontaneous deamination of 5‐methylcytosine (Figure S2h, Supporting Information). Although most patients had a tumor mutation burden comparable to that of patients with NSCLC in TCGA cohorts (Figure S2i, Supporting Information), the mutation counts were highly diverse, with a median value of 2.36 in log10 scale. Consistent with previous data from human patients,^[^ ^21^ ^]^ most of the SCLC malignant cells had somatic mutations in *TP53* (83%) and *RB1* (67%) (Figure S2j, Supporting Information). However, different SCLC samples had unique mutation genes. For example, mutations in the known tumor suppressors *FAT1* and *FAT4* occurred only in patients S1 and S5 (Figure S2j, Supporting Information). Taken together, our results indicate that SCLC malignant cells have high interpatient heterogeneity at both the genomic and transcriptomic levels and that the TME of metastatic SCLC in LNs is composed of diverse populations of malignant cells and immune cells.

### 2.2 Malignant Cells in Lymph‐Node Metastatic SCLC Have Inter‐Patient and Intra‐Tumor Heterogeneity Characterized by Distinct *ASCL1* and *NEUROD1* Expression Patterns

**Figure 2:**
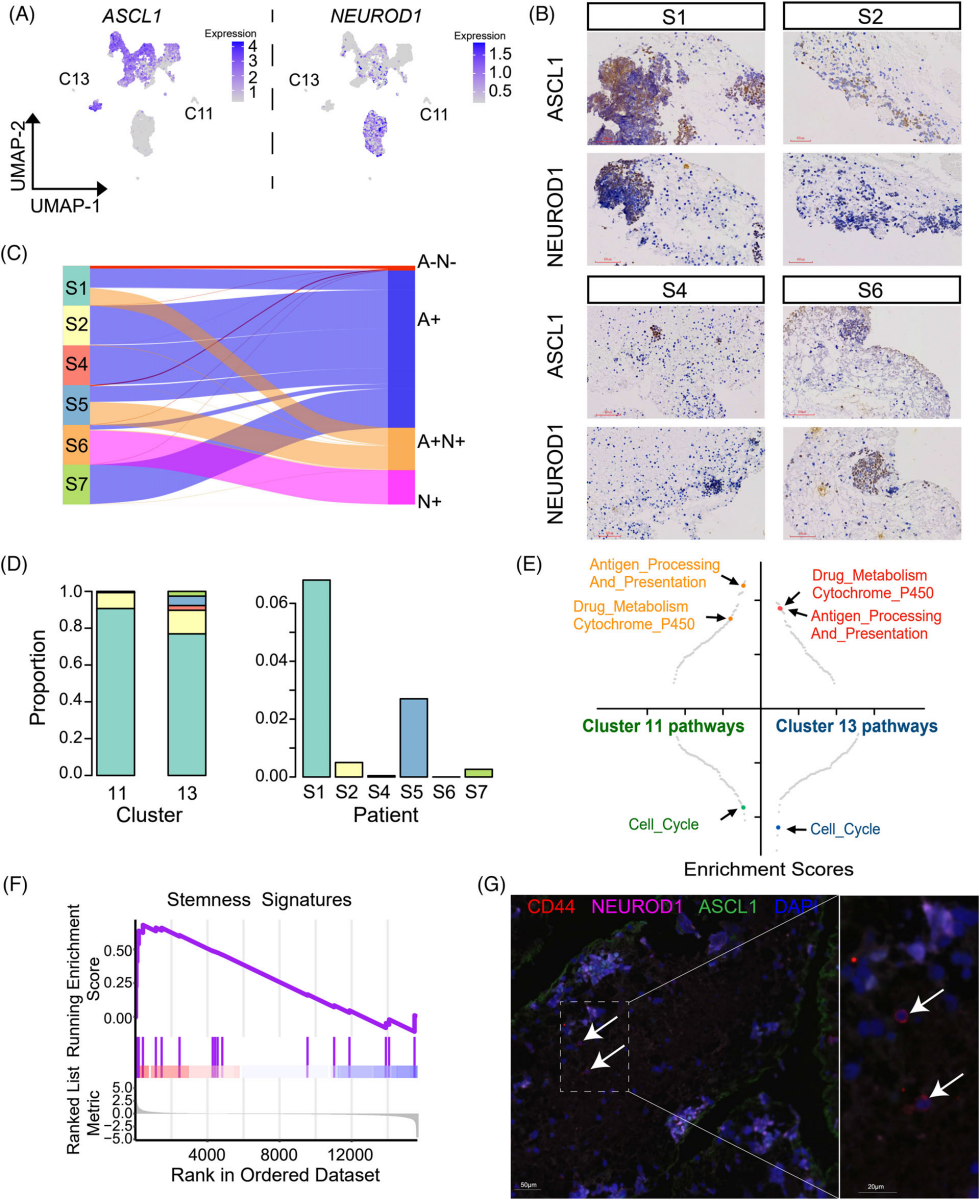
Malignant cells in lymph‐node metastatic SCLC have interpatient and intratumor heterogeneity. a) Visualization of *ASCL1* and *NEUROD1* expression in malignant cells by UMAP. b) Expression of *ASCL1* and *NEUROD1* in each patient, as assessed by IHC. c) Sankey diagram showing the expression patterns in 4 subpopulations of malignant cells, that is, *ASCL1*‐positive (A+), *NEUROD1‐*positive (N+), *ASCL1* and *NEUROD1* double positive (A+N+), and *ASCL1* and *NEUROD1* double negative (A‐N‐), in the 6 SCLC patients (without S3). d) Proportions of A‐N‐ malignant cells from different patients in clusters 11 and 13 (left panel); proportions of A‐N‐ malignant cells in each patient (right panel). e) GSEA revealing the pathways enriched in clusters 11 and 13. f) Stemness signatures enriched in cluster 11, as determined by GSEA. g) A representative multiplex immunohistochemistry image showing the expression of CD44 (red), ASCL1 (green), and NEUROD1 (pink) in EBUS sample of patient S1. Arrows point to CSC‐like cells (ASCL1‐ NEUROD1‐ CD44+).

Previous studies have classified SCLC subtypes according to the expression of four key transcription factors, that is, *ASCL1*, *NEUROD1*, *YAP1*, and *POU2F3*.^[^ ^20^ ^]^ To investigate SCLC subtypes at single‐cell level, we further re‐clustered all malignant cells except those from sample S3 (which had very few malignant cells) and obtained 14 subclusters (Figure S3a, Supporting Information). The NE markers *ASCL1* and/or *NEUROD1* were highly expressed in the malignant cells (**Figure** **2**a,b and Figure S3b, Supporting Information), while the expression of the non‐NE markers *YAP1* and *POU2F3* was rarely observed (Figures S3c,d and S4, Supporting Information). Then, we examined the expression of *ASCL1* and *NEUROD1* target genes, including their common targets *INSM1* and *HES6*; the *ASCL1*‐specific targets *MYCL* and *DLL3*; and the *NeuroD1*‐specific target *MYC*. The expression patterns of these genes were similar to that of *ASCL1* and *NEUROD1* respectively (Figure S3e, Supporting Information). We did not observe high expression of either *CD274* (PD‐L1) or *CTLA4* in malignant cells (Figure S3e, Supporting Information), consistent with previous findings that PD‐L1 expression was lower in SCLC than in other tumor types.^[^ ^22^ ^]^ Based on the expression of *ASCL1* and/or *NEUROD1*, all the malignant cells could be clustered into 4 subpopulations: 1) an *ASCL1*‐positive subpopulation (A+), 2) a *NEUROD1*‐positive subpopulation (N+), 3) a double‐positive (A+N+) subpopulation, and 4) a small population of a double‐negative (A‐N‐) subpopulation (Figure 2c). After comparison of the compositions of these subpopulations in each patient, we found that most malignant cells from patients S2, S4, and S7 had high expression of *ASCL1* (A+), while those from patient S6 were *NEUROD1*‐high (N+; Figure 2c and Figure S3f, Supporting Information). Malignant cells from patients S1 and S5 could be divided into A+ cells and those with high expression of both *ASCL1* and *NEUROD1* (A+N+; Figure 2c and Figure S3f, Supporting Information). To further distinguish these subpopulations, we analyzed the enriched pathways by gene set enrichment analysis (GSEA; Figure S5a,b, Supporting Information). The pathways related to cell cycle (green) and WNT (orange) were enriched in subpopulations with single‐positive A+ or N+ status compared to those with double‐positive A+N+ status (Figure S5a,b, Supporting Information). In addition, somatic mutations were frequently observed in the genes related to the WNT and cell cycle pathways (Figure S5c,d, Supporting Information).^[^ ^23^ ^]^ We also examined the copy number variations (CNVs) in all single cells using inferCNV. Consistent with the above findings, we observed frequently aberrant CNVs in genes related to the WNT and cell cycle pathways, such as *IRS2*, *TP53*, *RB1*, and *PTEN* (Figure S5e,f, Supporting Information). Our results suggested that WNT and cell cycle pathways may play important roles in, at least, a subset of SCLC cells.

Interestingly, A‐N‐ cells in clusters 11 and 13 were mainly from drug‐resistant tumors S1 and S2 (Figure 2d and Table S1, Supporting Information). Therefore, we hypothesized that the cells in clusters 11 and 13 may have the properties of tumor‐initiating cells. To test our hypothesis, we performed GSEA and found that both cluster 11 and cluster 13 had high expression of genes in the antigen processing and presentation and drug metabolism‐cytochrome P450 pathways and low expression of genes in the cell cycle pathway, compared to the other subpopulations (Figure 2e and Figure S5g, Supporting Information). We also observed that stemness signatures^[^ ^24^ ^]^ were enriched exclusively in cluster 11 (Figure 2f) but not in cluster 13. In particular, cluster 11 had high expression levels of cancer stem cell (CSC) marker genes,^[^ ^25^ ^]^ including *NOTCH1*, *KLF4*, *CD44*, *EPAS1*, and *MYC* (Figure S5h, Supporting Information). Consistently, immunohistochemical analysis of FFPE samples verified that a small proportion of CSC‐like cells (ASCL1‐ NEUROD1‐ CD44+) resided within SCLC tumors (Figure 2g). Our results suggested that a small number of A‐N‐ malignant cells had expression signatures of tumor‐initiating cells, which may provide therapeutic targets for SCLC.

Taken together, lymph‐node metastatic SCLC in each patient has mixed malignant cell types of A+, N+, A+N+, and A‐N‐ according to their expression level of NE markers *ASCL1* and/or *NEUROD1*. The expression profiles of different malignant cells may reflect their different roles during tumor progression. WNT and cell cycle pathways are enriched in A+ or N+ cells, whereas the expression signatures of tumor‐initiating cells are enriched in A‐N‐ cells.

### 2.3 High Expression of *FZD8* Was Correlated with Drug Resistance in SCLC Malignant Cells

**Figure 3:**
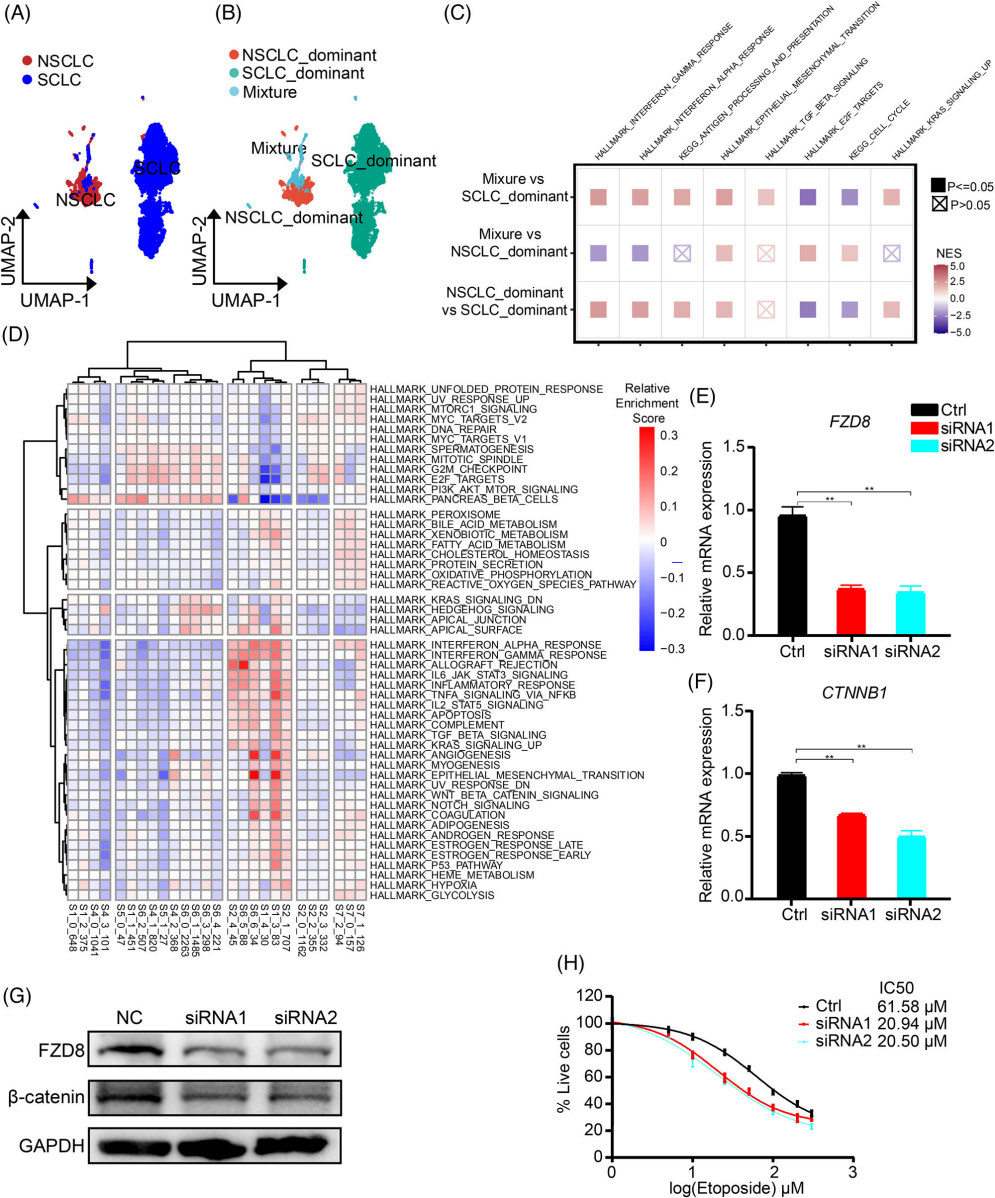
High expression of *FZD8* was correlated with drug resistance in SCLC malignant cells a) UMAP plots showing the malignant cells from lymph‐node metastatic SCLC and NSCLC patients. b) UMAP plots revealing three groups, that is, NSCLC dominant, SCLC dominant, and mixture groups of the malignant cells in (a). c) Comparison of the enriched pathways among the three groups. d) ssGSEA revealing different pathways between drug‐sensitive and resistant groups. e–h) After transfection of *FZD8* siRNAs (siRNA1 and 2) and control siRNA (Ctrl) in H446 cells, the mRNA level of e) *FZD8* and f) *CTNNB1* was examined by quantitative RT‐PCR (*n* = 3, ** *p* < 0.01). g) Protein levels of FZD8 and β‐catenin was assessed by Western blot (with GAPDH as internal control). h) Percentage of survived H446 cells after various concentration of etoposide treatment. Data were presented as Mean ± SD.

SCLC is quite different from NSCLC with regard to pathology and treatment. Especially immunotherapy did not work very well in SCLC compared with NSCLC.^[^ ^11^ ^]^ So we wondered whether their differences could be explained by different expression profiles. To match our samples, we chose the single‐cell transcriptome data of lymph‐node metastasis in NSCLC patients^[^ ^26^ ^]^ (**Figure** **3**a). Clearly, most lymph‐node metastatic malignant cells were clustered into either SCLC‐ or NSCLC‐dominant groups, although there was a mixture cluster that contains ≈4% of total SCLC cells and 19% of total NSCLC cells (Figure 3b). We compared the enriched pathways in SCLC‐dominant, NSCLC‐dominant, and mixture clusters (Figure 3c). Compared to NSCLC‐dominant cluster, SCLC‐dominant cluster had high expression of genes in cell cycle pathway but low expression of those in immune related pathways such as antigen presentation and interferon‐α and ‐γ responses, which may explain why SCLC malignant cells have high proliferation rate and low immune response. NSCLC malignant cells had higher expression of KRAS signaling pathway than SCLC. We also compared the difference in enriched pathways between drug‐ sensitive and resistant patients by standardized single sample GSEA (ssGSEA) (Figure 3d). We found that EMT, WNT, TGFβ pathways were enriched in malignant cells of drug‐resistant patients, while cell cycle pathway was not (Figure 3d). After analyzing the differentially expressed genes (DEGs) between drug sensitive and resistant patients, we found that *FZD8* in WNT signaling pathway had significantly high expression in the clones of drug resistant patients (Table S2, Supporting Information). To characterize its role in drug resistance, we knocked down *FZD8* in SCLC cell line H446 by siRNA (Figure 3e). As expected, the expression of downstream targets in WNT pathway such as *CTNNB1* was significantly reduced at mRNA levels (Figure 3f). We also validated the reduced protein level of β‐catenin (CTNNB1) due to depletion of *FZD8* (Figure 3g). Consequently, down‐regulation of *FZD8* resulted in sensitivity to etoposide (IC50 = 61.58 µm for control; IC50 = 20.94 and 20.50 µm respectively after siRNA1 and siRNA2 treatment; Figure 3h). Consistent with the findings by Wagner et al.,^[^ ^23^ ^]^ our results suggested that high expression of the essential genes such as *FZD8* in WNT pathway is highly correlated to drug resistance in SCLC malignant cells.

### 2.4 Immune Infiltration Is Related to Major Histocompatibility Complex Class I (MHC‐I)‐Related Gene Expression in Malignant Cells

**Figure 4:**
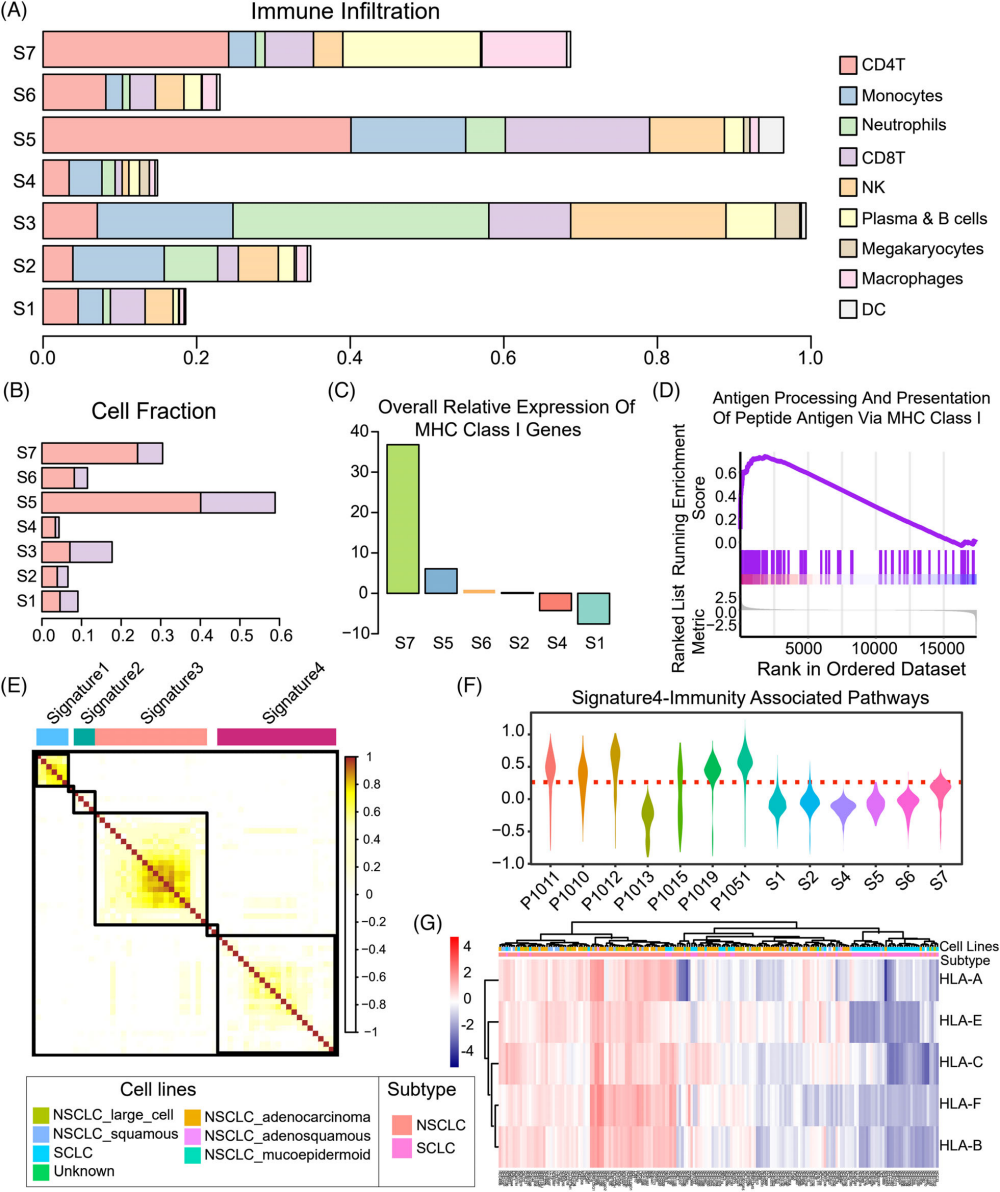
Immune infiltration is related to the expression of MHC‐I‐related genes in malignant cells. a) Proportions of different immune cells in each patient. b) Proportions of T cells in different patients. c) Relative expression of overall antigen processing‐related genes in each patient. d) GSEA showing that antigen processing and presentation of peptide antigens via MHC class I related genes were less activated in malignant cells of the patients with lower immune infiltration (S1, S2, S4, S6) than those with higher immune infiltration (S3, S5, S7). e) NMF analysis revealed four signatures between NSCLC and SCLC (Signature1, 2, and 4 are shown in Figure S6e, Supporting Information.). f) Immunity associated pathways of Signature4, including antigen processing and presentation of endogenous peptide antigen via MHC class I and positive regulation of T cell activation signaling, were less enriched in SCLC than NSCLC. g) Expression of MHC‐I genes in SCLC cell lines was lower than that in NSCLC based on the data from cancer cell line encyclopedia (CCLE).

As described above, SCLC malignant cells had dysfunction in antigen presentation. Therefore, we speculated that the immune cell infiltration will be affected. We examined the immune cells in SCLC at the single‐cell level. First, the composition of different immune cell types varied greatly among the 7 SCLC samples (**Figure** **4**a). Patients S1, S2, S4, and S6 had less infiltration of immune cells, especially T cells, than the other patients (Figure 4a,b), which may explain why some SCLC patients fail to benefit from immunotherapy.^[^ ^12^, ^14^ ^]^ To understand the interaction between malignant cells and immune infiltration in SCLC, we analyzed the antigen presenting genes in the malignant cells from each patient. As expected, the overall expression of MHC‐I related genes in the malignant cells was lower in patients with less immune cell infiltration (Figure 4c and Figure S6a, Supporting Information). Although SCLC has a relatively high mutation burden as shown by previous findings^[^ ^27^ ^]^ and our results (Figure S2i, Supporting Information), genomic mutations may not contribute to dysregulation of these genes since there were very few mutations related to MHC‐I antigen presentation (Figure S6b, Supporting Information). Moreover, GSEA results showed that the pathways associated with antigen processing via MHC‐I molecules and response to type I Interferon were enriched in the malignant cells of SCLC samples with high immune infiltration levels (Figure 4d and Figure S6c,d, Supporting Information). To further extract SCLC expression features in comparison to NSCLC, we performed dimension reduction analyses, that is, nonnegative matrix factorization (NMF). We identified a total of 56 co‐expressed metagenes in clusters of malignant cells across SCLC and NSCLC tumors and extracted 6 signatures (two of which were not described in detail due to very few metagenes in them) that distinguished SCLC from NSCLC (Figure 4e and Table S3, Supporting Information). Consistently, the signature of immune associated pathways (signature4 in Figure 4f) was markedly down‐regulated in SCLC malignant cells, whereas the signature of cell cycle (signature1 in Figure S6e, Supporting Information) was up‐regulated. Indeed, MHC‐I genes were down‐regulated in SCLC cell lines compared to NSCLC according to cancer cell line encyclopedia (CCLE) (Figure 4g and Figure S6f, Supporting Information). Collectively, our results indicated that immune infiltration in SCLC is associated with MHC‐I antigen presentation of malignant cells in SCLC.

### 2.5 Exhausted CD8+ T Cells Are Not Abundant in SCLC Patients

**Figure 5:**
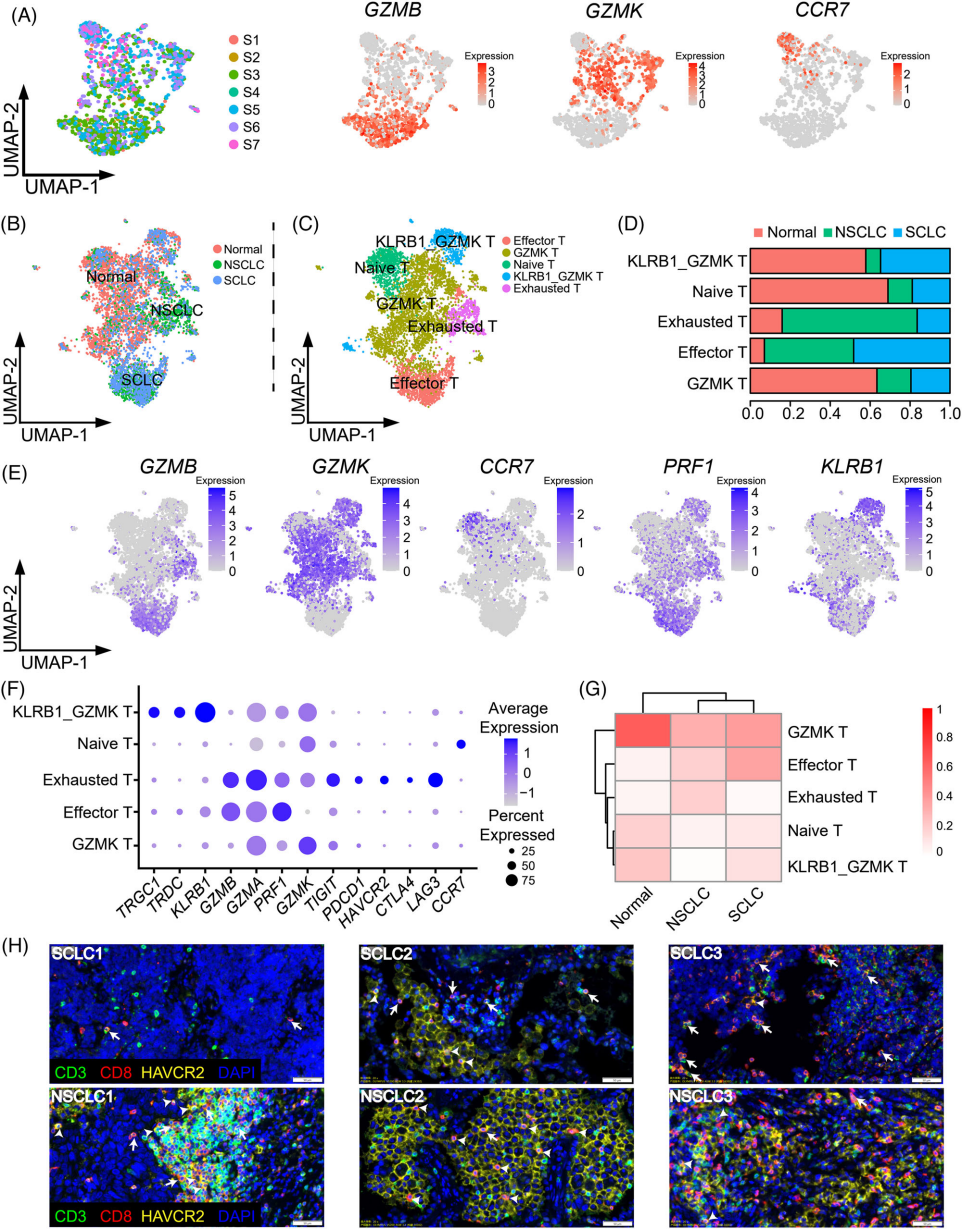
Exhausted CD8+ T cells are not abundant in SCLC patients. a) UMAP plots showing the expression of *GZMB*, *GZMK*, and *CCR7* in CD8+ T cells. b) UMAP plots showing the CD8+ T cells from SCLC, NSCLC, and normal lymph nodes. c) UMAP plots revealing the CD8+ T cells from different patients can be divided into 5 groups (effector, GZMK, naïve, KLRB1_GZMK, and exhausted T cells). d) The proportion of SCLC, NSCLC, and normal CD8+ T cells in effector, GZMK, naïve, KLRB1_GZMK, and exhausted T groups. e) The expression of *GZMB*, *GZMK*, *CCR7*, *PRF1*, *KLRB1* in CD8+ T cells was displayed via UMAP. f) The expression of *TRGC1*, *TRDC*, *KLRB1*, *GZMB*, *GZMA*, *PRF1*, *GZMK*, *TIGIT*, *PDCD1*, *HAVCR2*, *CTLA4*, *LAG3*, and *CCR7* in CD8+ T cells was displayed. g) Proportions of effector, GZMK, naïve, KLRB1_GZMK, and exhausted T cells in SCLC, NSCLC, and normal CD8+ T cells. h) Representative multiplex immunohistochemistry images showing SCLC samples had less HAVCR2+ CD8+T cells (CD3+ (green), CD8+ (red) and HAVCR2+ (yellow)) than NSCLC. Arrows point to HAVCR2‐ CD8+T cells (CD3+ CD8+), while arrowheads point to HAVCR2+ CD8+ T cells (CD3+ CD8+ HAVCR2+).

As dysfunction of antigen presentation in malignant cells may hinder the cytotoxicity attack by CD8+ T cells, we examined the CD8+ T cell populations within the SCLC TME. Overall, CD8+ T cells had distinct gene expression patterns of *GZMB*, *GZMK*, and *CCR7* (**Figure** **5**a). Both GZMB+ and GZMK+ cells had high expression of GZMA (Figure S7a, Supporting Information). Neither population had high expression of known markers of immune exhaustion such as *LAG3*, *HAVCR2*, *CTLA4*, and *PDCD1* (Figure S7a, Supporting Information). To investigate the difference in CD8+ T cells between SCLC and NSCLC, we compared our data with the other's.^[^ ^26^ ^]^ Even after elimination of batch‐effect by Harmony, CD8+ T cells from SCLC and NSCLC were overlapped, suggesting that they overall have similar expression patterns (Figure 5b). According to their differential expression of marker genes, CD8+ T cells can be grouped into effector, GZMK, naïve, KLRB1_GZMK, and exhausted T cells (Figure 5c,d). Effector T cells have high expression of *GZMB*, *GZMA*, *PRF1* but not *GZMK*. GZMK T cells exclusively have high expression of *GZMK* and *GZMA* but not other granzymes. KLRB1_GZMK T cells have high expression of *KLRB1* and *GZMK* in addition to TCR related genes such as *TRGC1* and *TRDC*. Exhausted T cells highly express *TIGIT*, *LAG3*, *CTLA4*, *PDCD1*, and *HAVCR2*. Naïve T cells highly express *CCR7* (Figure 5e,f and Figure S7b, Supporting Information). Compared to those in NSCLC, CD8+ T cells in SCLC consisted of more effector T cells but less exhausted T cells (Figure 5g). To verify the bioinformatic analysis, we performed multiplex immunohistochemistry of additional SCLC samples with NSCLC samples as control. Consistently, SCLC samples had less exhausted CD8+ T cells (CD3+ CD8+ HAVCR2+) than NSCLC (Figure 5h). In conclusion, most CD8+ T cells in SCLC are effective but allow SCLC malignant cells to evade cytotoxicity most likely due to their dysfunction of antigen presentation.

### 2.6 NK Cells in SCLC Have Impaired Cytotoxicity Which Is Correlated with Their High Expression of *TGFBR2*

**Figure 6:**
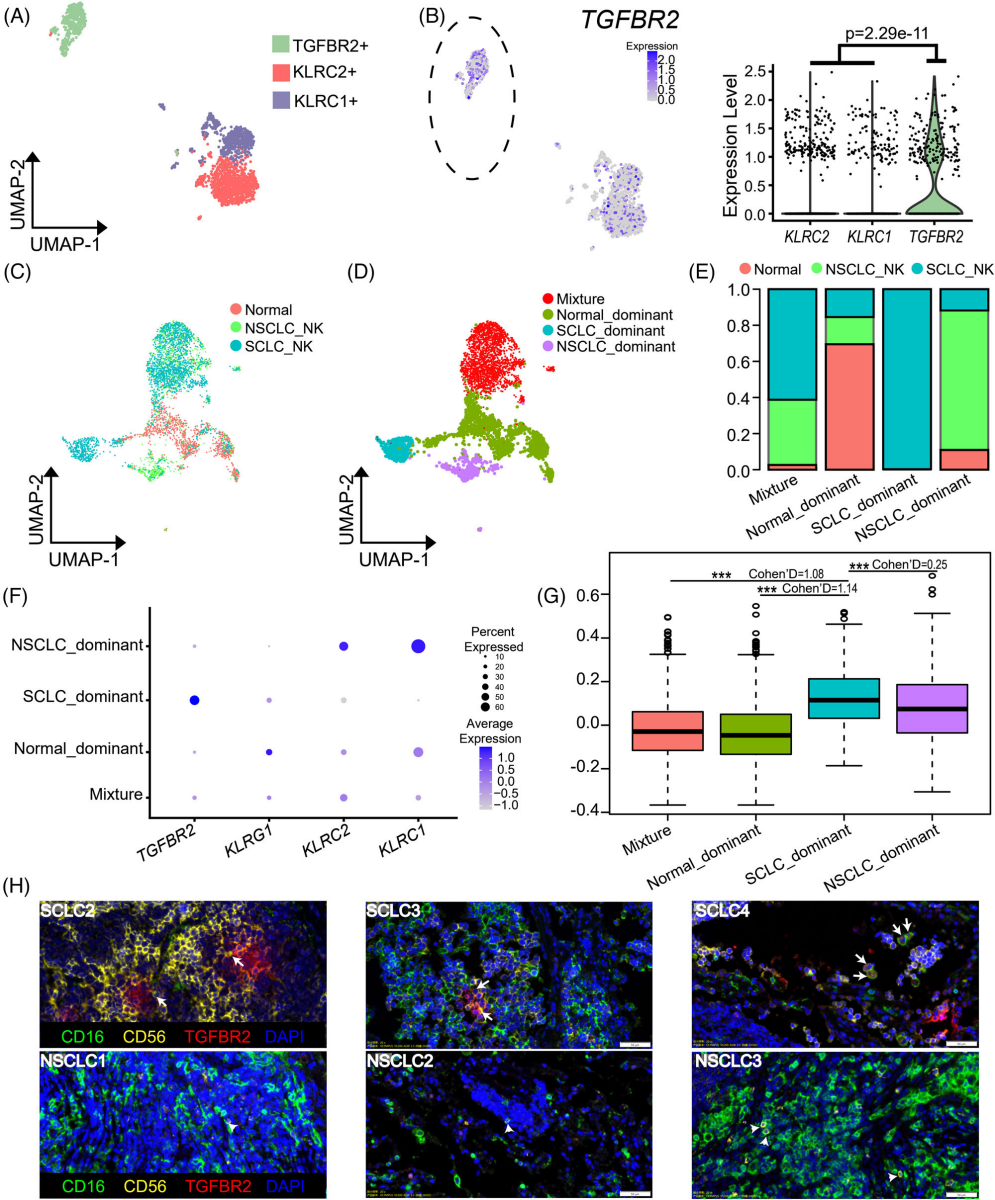
NK cells in SCLC patients have impaired cytotoxicity. a) UMAP plot showing 3 clusters of NK cells based on differential expression of *TGFBR2*, *KLRC1*, and *KLRC2*. b) UMAP plots showing the TGFBR2+ cluster (left panels); violin plot revealing the higher expression of *TGFBR2* (*p* = 2.29e‐11) in TGFBR2+ cluster (right panels). c) UMAP plots showing all the NK cells from SCLC, NSCLC, and normal controls. d) UMAP plots revealing four groups of NK cells, that is, mixture, normal dominant, SCLC dominant, NSCLC dominant. e) Proportions of SCLC‐, NSCLC‐ and normal‐NK cells in the mixture, normal‐dominant, SCLC‐dominant, NSCLC‐dominant NK subgroups. f) The expression of *TGFBR2*, *KLRG1*, *KLRC2*, and *KLRC1* in different NK subgroups. g) TGF‐β response signatures of SCLC‐dominant NK subgroup were higher than those of the others respectively (SCLC‐dominant vs mixture, *p* < 2.2e‐16, Cohen's D = 1.08; vs normal‐dominant, *p* < 2.2e‐16, Cohen's D = 1.14; vs NSCLC‐dominant, *p* = 0.0008, Cohen's D = 0.25). h) Representative multiplex immunohistochemistry images showing TGFBR2+ NK cells (CD16+ (green), CD56+ (yellow) and TGFBR2+ (red)) are predominantly in SCLC, compared to NSCLC. Arrows point to TGFBR2+ NK cells (CD16+ CD56+ TGFBR2+), while arrowheads point to TGFBR2‐ NK cells (CD16+ CD56+).

NK cells play important roles in the defense against microbial infection and tumors. Their function is regulated by the expression of different activating and inhibitory receptors. Based on their expression profiles, NK cells in SCLC patients can be basically categorized into three clusters. Two of them had high expression of either killer cell lectin like receptor C1 (*KLRC1*) or *KLRC2*, which were referred to as KLRC1+ and KLRC2+ respectively (**Figure** **6**a). The top expressed genes in another cluster of cells included *FCGR3B*, *CXCR2*, *CXCR1*, and *TGFBR2*. However, *FCGR3B* encodes CD16 which is a common NK cell marker. *CXCR2* and *CXCR1* are expressed in multiple lineages of immune cells. TGFBR2 is associated with the activation of TGF‐β signaling pathway. Best et al.^[^ ^28^ ^]^ showed that TGF‐β signaling activation in NK cells contributes to the metastasis of SCLC. Therefore, we selected *TGFBR2* as the major feature for this cluster (TGFBR2+; Figure 6b). *KLRC1* and *KLRC2* encode inhibitory and activating receptors, respectively. KLRC1+ cluster had high expression of *KLRC1* and the known exhaustion marker *HAVCR2* (Figure S8a,b, Supporting Information), indicating their exhaustion status. Although it expressed the activating receptor, KLRC2+ cluster also had high expression of the known exhaustion gene *TIGIT* (Figure S8c,d, Supporting Information), suggesting that like KLRC1+ NK cells, KLRC2+ NK cells may lose antitumor activity. Notably, TGFBR2+ NK cells were the most immunosuppressive population, since they had significantly lower expression of *GZMB* (*p* < 2.2e‐16) and *PRF1* (*p* < 2.2e‐16) than KLRC1+ and KLRC2+ clusters (Figure S8e, Supporting Information), consistent with a recent study.^[^ ^28^ ^]^ To determine the difference of NK cells between SCLC and NSCLC, we pooled the NK cell data from our SCLC dataset and a previously published NSCLC dataset (Figure 6c). Indeed, the NK cells from SCLC and NSCLC could be grouped into distinct clusters according to their gene expression signatures, that is, normal‐dominant, SCLC‐dominant, NSCLC‐dominant, and mixture subpopulations (Figure 6d, e). Although they had relatively high expression of the cytotoxicity markers such as *PRF1*, *GZMA*, and *GZMB* except *GZMK* (Figure S8f,g, Supporting Information), NK cells in SCLC‐dominant subpopulation were most likely exhausted as they had high expression of *TGFBR2* and low expression of *KLRC2* (Figure 6f). Consistently, TGF‐β response signatures of SCLC‐dominant group were significantly higher than those of the others (Figure 6g). To further confirm *TGFBR2* expression pattern in SCLC, we collected another three SCLC and three NSCLC samples respectively and performed multiplex immunohistochemistry. Indeed, there were more TGFBR2+ NK cells (CD16+ CD56+ TGFBR2+) in SCLC than NSCLC (Figure 6h and Figure S8h, Supporting Information). Taken together, these findings indicate that high expression of *TGFBR2* in SCLC‐dominant NK cells can impair their cytotoxic activity,^[^ ^29^ ^]^ favoring tumor growth and metastasis.

## 3 Discussion

In this study, we intensively analyzed the heterogeneity of human lymph‐node metastatic SCLC at the single‐cell level. Rudin et al. summarized the previous nomenclature of SCLC subtypes and proposed categorizing SCLC into the SCLC‐A, SCLC‐N, SCLC‐Y, and SCLC‐P subtypes based on the expression of *ASCL1*, *NEUROD1*, *YAP1*, and *POU2F3*, respectively.^[^ ^20^ ^]^ Among 81 primary tumors,^[^ ^3a^ ^]^ SCLC‐A was found to be the most common subtype, followed by SCLC‐N, SCLC‐Y, and SCLC‐P. However, the subtypes were classified based on bulk expression data. At the single‐cell level, we first identified a new subtype of SCLC malignant cells with expression of both *ASCL1* and *NEUROD1* (i.e., A+N+ cells). The genes related to cell cycle and WNT pathways were respectively enriched in A+ or N+ malignant cells compared to A+N+ malignant cells, which is also consistent with the somatic mutations found in these pathways. Somatic mutation and transcriptional upregulation of the WNT pathway are associated with chemorefractoriness in SCLC.^[^ ^23^ ^]^ By integrated analysis, we provided evidence that high expression of *FZD8* in WNT pathway was correlated with drug resistance in SCLC malignant cells.^[^ ^30^ ^]^ Second, *ASCL1* and *NEUROD1* were indeed the most common markers in the malignant cells from the SCLC patients. However, certain cell types (i.e., A+, N+, and A+N+) are dominant in a given tumor tissue and can be mixed with other types. It would be interesting to further investigate the temporal transition caused by key regulators such as MYC as reported previously,^[^ ^31^ ^]^ which may require multiple sampling during a time course. Third, we characterized a small number of A‐N‐ cells in patients with poor prognosis. A‐N‐ cells (cluster 11) are most likely dormant tumor‐initiating cells with relatively low expression of genes in the cell cycle pathway and high expression of those in the antigen processing/presentation and drug metabolism‐cytochrome P450 pathways. Dysregulation of these pathways in A‐N‐ cells may promote a CSC‐like phenotype. Thus, targeting A‐N‐ cells may lead to enhanced therapeutic efficacy in SCLC. Consistent with recent findings in eight CDX models,^[^ ^19^ ^]^ we did not observe distinct cell clusters with high expression of either *POU2F3* or *YAP1*, most likely because of our limited sample size. Our work made a concerted attempt to enhance the understanding of SCLC subtypes at the single‐cell level and provided insight into new biomarkers or therapeutics targeting specific SCLC cell types.

US FDA approval of atezolizumab (an anti‐PD‐L1 antibody) in combination with platinum‐based chemotherapy for the first‐line treatment of ES‐SCLC was an important milestone.^[^ ^12^ ^]^ Subsequently, a combination of durvalumab (an anti‐PD‐L1 antibody) and chemotherapy entered phase 3 trials and was found to similarly improve the OS of the patients.^[^ ^32^ ^]^ Unfortunately, however, CheckMate trials 331 and 451 of nivolumab (an anti‐PD1 antibody) did not show positive results.^[^ ^33^ ^]^ The results of these trials stimulated our intense curiosity about immune infiltration in the TME of SCLC. LNs are the central hubs for recirculating immune cells^[^ ^34^ ^]^ and frequent sites of SCLC metastasis. However, little is known about the immune environment in lymph‐node metastatic SCLC. As we retrieved different cell types from EBUS biopsies, we first examined the malignant cells and their expression patterns of genes related to immune infiltration. We found that malignant cells create an immunosuppressive environment by downregulating MHC‐I‐related genes and that low expression of MHC‐I‐related genes is probably independent of somatic mutations. Interestingly, CD4+ and CD8+ T cell infiltration is associated with the expression of MHC‐I‐related genes which provides evidence at the single‐cell level to support the use of immunotherapies targeting T cells. We then examined T cells, especially CD8+ T cells, which had distinct gene expression patterns of *GZMB*, *GZMK*, and *CCR7*. Integrated analysis showed that CD8+ T cells in SCLC are less exhausted. In another integrated scRNA‐seq analysis,^[^ ^35^ ^]^ however, high expression of exhaustion markers was found in CD8+ T cells of NSCLC samples. This may explain why immunotherapy targeting CD8+ T cells did not work well for SCLC patients compared to NSCLC. Meanwhile, our results shed light on new mechanisms for the development of novel immunotherapies for SCLC. New approaches have been proposed to target innate immune cells such as macrophages and NK cells.^[^ ^5^ ^]^ NK cells in EBUS biopsies were abundant but exhibited impaired antitumor function. KLRC2+ NK cell cluster, which should be an antitumor subtype, showed an immunosuppressive phenotype with high expression of *TIGIT* (Figure S8d, Supporting Information). Immunosuppressive NK cells included KLRC1+ and TGFBR2+ clusters. Especially, TGFBR2+ cluster was more abundant in SCLC than NSCLC (Figure 6f). Knockout *TGFBR2* in NK cells can enhance their in‐vitro and in‐vivo cytotoxic effect on lung cancer^[^ ^36^ ^]^ and glioblastoma.^[^ ^37^ ^]^ In addition, blockade of the TGF‐β signaling pathway can inhibit SCLC metastasis.^[^ ^28^ ^]^ This implied that immunotherapy targeting TGFBR2+ NK cells may offer a promising treatment of SCLC by killing and preventing metastasis of malignant cells. Taken together, our findings sketch a picture of the diverse immune environment in SCLC, which might explain the differing efficacy of immune checkpoint blockade therapies among patients in several clinical trials,^[^ ^38^ ^]^ and highlight the immune cells and their characteristic markers with potential utility for new treatments.

Minimally invasive methods such as CT guided biopsy, mediastinoscopy, and EBUS‐TBNA provide accurate diagnosis and staging for lung cancers. Our results indicate that EBUS‐TBNA is accurate for diagnosing SCLC and provides cells with sufficiently high quality for scRNA‐seq. Since it was established in the 1990s,^[^ ^39^ ^]^ EBUS‐TBNA has been widely accepted in lung cancer diagnostics due to its efficacy and safety compared to other minimally invasive methods. EBUS‐TBNA is performed using a dedicated bronchoscope equipped with an ultrasound transducer. This technique decreases the risk of hemorrhea or pneumothorax^[^ ^40^ ^]^ and not only supports direct observation of regional lesions but also allows excellent access to the targeted mass in the peribronchial/peritracheal region.^[^ ^41^ ^]^ In this work, we demonstrated that samples collected by EBUS‐TBNA showed an accurate diagnosis of SCLC at the single‐cell level. Notably, however, the sample from patient S3 contained few malignant cells, implying that EBUS may sometimes be insufficient for pathological evaluation. As stated above, lymph‐node metastatic SCLC tumors retain NE signatures with high expression of *ASCL1* and *NEUROD1*, at least in our samples, although whether the signatures are altered during metastasis needs to be elucidated.

## 4 Experimental Section

### Patients and Samples

A total of seven patients with metastatic SCLC in mediastinal LN were enrolled between May 9, 2019 and Jan 8, 2020. EBUS‐TBNA was performed with diagnostic intent. All EBUS‐TBNA procedures were done in the operating room with anesthesia using a set of equipment including EBUS probe (BF‐UC180F; Olympus, Tokyo, Japan), fiberoptic bronchoscope (BF‐UC260FW; Olympus, Tokyo, Japan), and 22‐guage needle (NA‐201SX‐4022; Olympus, Tokyo, Japan). After operation, the samples were prepared for cytological examination, hematoxylin and eosin examination, and immunohistochemistry. Moreover, 5 mL whole blood was collected for further genomic analyses. This work was approved by the Ethical Committee for Clinical Research of Shanghai Pulmonary hospital under approval number K20‐434. The informed consent of each patient was obtained before the operation.

### Preparation of Single‐Cell Suspensions

EBUS‐TBNA samples were stored in iced‐DMEM/F12 before they were divided into three aliquots, one for single‐cell suspensions, one fixed by 4% formaldehyde for immunohistochemistry, the other frozen by liquid nitrogen for genomic sequencing. Single‐cell suspensions were then made within 2 h after EBUS‐TBNA. Briefly, the samples were transported to the lab and rinsed with cold phosphate buffered saline (PBS, Hyclone, USA) containing 10% fetal bovine serum (FBS, Gibco, USA) and 1% penicillin/streptomycin (Gibco, USA). Then they were digested with 10 mL digestion medium containing 0.2% collagenase II (ThermoFisher Scientific, USA), 400 U mL^−1^ DNase I (Roche, Switzerland) in DMEM/F12. After incubation at 37 °C for 15 min, the samples were vortexed for 10 s and pipetted up and down for 1 min with pipettes of descending sizes. The samples were filtered through a 70‐µm nylon mesh after addition of 20 mL ice‐cold PBS containing 10% FBS. After centrifugation at 100 × *g* and 4 °C for 5 min, the supernatant was discarded and the cell pellet was resuspended in 2 mL 1X BD Pharma Lyse (BD Biosciences, USA) and incubated at room temperature for 10 min. The cell pellet was collected by centrifuging at 100 × *g* and 4 °C for 5 min and resuspended in 1 mL ice‐cold PBS containing 2% FBS. 10 µL of the cell suspension was counted by an automated cell counter (Luna, ThermoFisher Scientific, USA) to determine the concentration of live cells.

### Droplet‐Based scRNA‐seq

An estimated 8,000 cells for each sample were loaded to 10X Genomics Chromium instrument to generate cDNA libraries by using Chromium Single Cell 3′ Reagent Kits (10X Genomics, USA) following the manufacturer's instructions. The libraries were sequenced by Illumina Hiseq X Ten platform.

### Single‐Cell RNA Sequencing Analysis

The scRNA‐seq data of metastatic NSCLC LN and normal LN were obtained from previous study.^[^ ^26^ ^]^ Both SCLC and NSCLC sequence reads were mapped to the hg38 reference genome and the corresponding unique molecular identified (UMI) were qualified through the cell ranger workflow (version 3.1.0). Cells with low UMI counts identified were preliminarily filtered in the workflow. The ambient RNA contamination was assessed and removed using SoupX (version 1.5.2) with default parameters.^[^ ^42^ ^]^ The corrected UMI count matrix derived from SoupX was further analyzed using Seurat package (version 4.0.6) in R program.^[^ ^43^ ^]^ Meanwhile, cells with any following criteria were additionally excluded: 1) Cells with extreme feature counts (<500 or > 10 000); 2) >20% reads aligned to mitochondria; 3) Cells with extreme RNA counts (<1800 or >100 000). DoubletFinder (version 2.0.3) was used to identify potential doublet cells.^[^ ^42^, ^44^ ^]^ Total 850 putative doublets were excluded from further analysis. Harmony algorithm was used to correct the potential confounders when integrating the scRNA‐seq data from multiple samples. Specifically, for integration of SCLC samples, harmony was used to correct the covariable of sample ID. For the integration analysis of SCLC and NSCLC samples, covariates of both tumor type and sample ID were corrected.

After filtering of low‐quality cells, the UMI count matrix was normalized by the *NormalizeData* function in Seurat, and highly variable features were calculated using the *FindVariableFeatures* function. Principal component analysis (PCA) was performed on linear transformation scaled data to reduce noise. Uniform Manifold Approximation and Projection (UMAP) was facilitated for dimension reduction with setting the parameter *reduction* as “harmony,” *metric* as “correlation” and *umap.method* as “umap‐learn.” *FindNeighbors* and *FindClusters* function were applied to detect communities and find cell clusters.

The *FindAllMarkers* function in Seurat was used to find markers for each of the identified clusters. Differentially expressed genes between drug sensitive resistant (S1 and S2) and drug sensitive (S4, S5, and S7) patients were found by *FindMarkers* function using the default Wilcoxon rank sum test

### Cell Annotation

Cell type annotations were implemented according to the expression of the canonical cell markers and by inspecting the differentially expressed genes of each cluster. The identity of each cluster was further corroborated using the PanglaoDB database (https://panglaodb.se).^[^ ^18^ ^]^

### CNV Inferred from scRNA‐seq

Large‐scale chromosomal CNVs within the cancer cells at single‐cell level were identified by InferCNV^[^ ^45^ ^]^ (https://github.com/broadinstitute/inferCNV). Global gene expression of the cancer cells was compared with the non‐malignant cells during CNV inference. Bayesian Network Latent Mixture Model in the inferCNV was used to infer the variation degrees. Output values of 0.5, 1, 1.5 were referred to as copy number deletion, neutral, and copy number amplification respectively. Malignant cells have been clustered separately according to samples by setting “cluster_by_groups = T.”

### NMF Analysis

To reveal the transcriptional heterogeneity of NSCLC and SCLC malignant cells, variably expressed metagenes and gene expression signatures were identified by NMF analysis. NMF R package was used for the unsupervised NMF analysis.^[^ ^46^ ^]^ A total of 56 metagenes were identified across the 14 tumors. Within one sample, 100 top‐ranked genes with highest NMF loadings of each metagene were selected. The 56 metagenes were then grouped by hierarchical clustering, using one minus the Pearson correlation coefficient over loading scores of selected genes as a distance metric. Four clusters of signatures were identified manually. Clusters with metagene counts fewer than 4 were not considered.

### GSEA and ssGSEA

GSEA was used to determine the enrichment of the gene sets and to assess the significance. The enrichment patterns were illustrated by the ClusterProfiler R package. To further explore the pathways associated with chemotherapy resistance, ssGSEA method from R software Gene Set Variation Analysis (GSVA) package was applied.^[^ ^47^ ^]^ Gene sets belonging to Gene Ontology (GO, V7.1), biological process, and Enriched Kyoto Encyclopedia of Genes and Genomes (KEGG, V7.1) pathway were used to annotate the gene sets.

### Cell Culture and Cell Viability Assay

H446 SCLC cell lines were cultured in RPMI 1640 medium with 10% FBS (Gibco) and 1% penicillin‐streptomycin (Gibco). H446 cells transfected with siRNA were seeded into 96‐well plates (5000 cells well^−1^) in triplicate. Then cells were treated with drugs at various concentrations. After 48 h incubation, cell viability was detected using cell counting kit‐8 (Dojindo). Finally, normalized data and transformed concentrations were analyzed to determine IC50 using GraphPad Prism (GraphPad Software Inc.; USA).

### CCLE

CCLE (https://sites.broadinstitute.org/ccle) was used to validate the expression of MHC I class related genes in SCLC and NSCLC cell lines. In current study, 51 SCLC and 137 NSCLC cell lines were applied for the analysis.

### WES and Analysis

Genomic DNA (gDNA) from peripheral blood was extracted by TIANamp Blood DNA Midi Kit (TIANGEN, China). gDNA from the matched tumor samples was extracted by QIAamp DNA Micro Kit (Qiagen, Germany). After DNA qualification, the sequencing libraries were constructed by Agilent SureSelect Human All Exon V6 kit (Agilent Technologies, USA). Sequencing was done by Illumina Novaseq 6000 platform (Illumina Inc., San Diego, USA) in Novogene Bioinformatics Technology Co., Ltd (Beijing, China). Finally, the ≈150 bp paired‐end reads were generated with a minimum coverage of 10× for ≈99% of the human genome (mean coverage was 100× for gDNA from peripheral blood, and 150× for gDNA from frozen tumor sample).

The somatic mutations were called by Mutect2 following GATK's best practice pipeline (https://gatk.broadinstitute.org/hc/en‐us/articles/360035894731). FASTQ data were aligned to human genome reference (hg19_b37) by Burrows‐Wheeler Aligner (BWA),^[^ ^48^ ^]^ followed by marking duplicates, Base Quality Score Recalibration. The candidate variants were called and filtered through estimated Contamination and Orientation Bias. Somatic mutations passing the filters were then annotated by funcotator.

### Mutational Signature Analysis

To detect mutational signature, non‐negative matrix factorization (NMF) approach from R package SomaticSignatures was applied. Mutation data from WES was converted into matrix composed of 96 mutational features caused by six base substitutions (C > A, C > T, C > G, T > A, T > G, and T > C). To better analyze the signatures, identified signatures were compared with known COSMIC cancer signatures (http://cancer.sanger.ac.uk/cosmic/signatures). Cosine similarity analysis was calculated to discover the best match.

### Immunostaining (IHC) and Multiplex Immunohistochemistry

The 4 mm thick FFPE tissues were deparaffinized in xylene before they were rehydrated in gradient concentrations of ethanol. In antigen retrieval step, the samples were incubated in sodium citrate buffer (PH = 6.0) for 4 min. Hydrogen peroxide was used to block the endogenous peroxidase activity for 10 min. The samples were covered with the primary antibodies (ASCL1, Abcam, ab213151; NeuorD1, Abcam, ab60704; YAP1, CST, 14074; POU2F3, Abcam, ab191840; CD44, CST, 3570). After incubation with the secondary antibodies for IHC, the samples were dehydrated, covered with coverslips as previously done.^[^ ^49^ ^]^ For multiplex immunohistochemistry, PANO 4‐plex IHC kit (Panovue, Beijing, China) was used. Panel 1 antibodies (CD3, Abcam, ab17143; CD8, CST, 70306; HAVCR2, CST, 45208), and panel 2 antibodies (CD16, Abcam, ab246222; CD56, CST, 99746S; TGFBR2, Abcam, ab78419) were applied sequentially. And Mantra System (PerkinElmer, Waltham, Massachusetts, US) was utilized to obtain multispectral images.

### Statistical Analysis

The distribution of Geni index between tumor cells and non‐tumor cells was compared by two‐sided Wilcoxon rank‐sum test using the ggsignif R package. The difference between the experimental and the control groups in cell lines was determined by two‐sided Student's *t*‐test using stats R package (*n* = 3). The expression difference of MHC class I associated genes between NSCLC and SCLC cell lines in the CCLE database was examined by two‐sided Student's *t*‐test using the stats R package. Comparisons of expression differences of selected genes among the three NK cell subgroups were conducted by two‐sided Student's *t*‐test using the stats R package, and Cohen's D value was calculated by the effectsize R package. The Seurat *FindMarkers* function was applied to identify differential expressed genes between drug resistant and drug sensitive samples under the default Wilcoxon rank sum test. Significant differential expressed genes were determined based on the statistical threshold (log2 fold change > 0.25, *p*‐value < 0.01, and Bonferroni corrected *p*‐value < 0.01). GraphPad Prism 7.0 and R version 4.1.2 software were used to depict the pictures. *P* value less than 0.05 was considered as statistically significant.

## Conflict of Interest

The authors declare no conflict of interest.

## Ethics Statement

This work was approved by the Ethical Committee for Clinical Research of Shanghai Pulmonary hospital under approval number K20‐434. The informed consent of each patient was obtained before the operation.

## Supporting information

Supporting Information

Supplemental Table 1

Supplemental Table 2

Supplemental Table 3

## Data Availability

The raw sequence data reported in this paper have been deposited in the Genome Sequence Archive^[^ ^50^ ^]^ in National Genomics Data Center,^[^ ^51^ ^]^ China National Center for Bioinformation/Beijing Institute of Genomics, Chinese Academy of Sciences, under accession number HRA000607 that are publicly accessible at http://bigd.big.ac.cn/gsa-human. The scripts which were used to analyze the data have been prepared as readable jupyter notebook files and uploaded to GitHub (https://github.com/DiankeLi/mSCLC).
