# Supplementary material for: Single‐Cell Transcriptome Identifies Drug‐Resistance Signature and Immunosuppressive Microenvironment in Metastatic Small Cell Lung Cancer
Source: Adv Genet (Hoboken). 2022 Mar 4;3(2):2100060. doi: 10.1002/ggn2.202100060 (PMC9744506; doi:10.1002/ggn2.202100060)
Supplement: Supplementary file 1 — Supporting Information [file GGN2-3-2100060-s002.pdf]

ADVANCED GENETICS
Open Access

Supporting Information

for *Advanced Genetics*, DOI 10.1002/ggn2.202100060

Single-Cell Transcriptome Identifies Drug-Resistance Signature and Immunosuppressive Microenvironment in Metastatic Small Cell Lung Cancer

Jing Zhang, Haiping Zhang, Lele Zhang, Dianke Li, Mengfan Qi, Liping Zhang, Huansha Yu, Di Wang, Gening Jiang, Xujun Wang, Xianmin Zhu* and Peng Zhang**

Supplementary figure legend

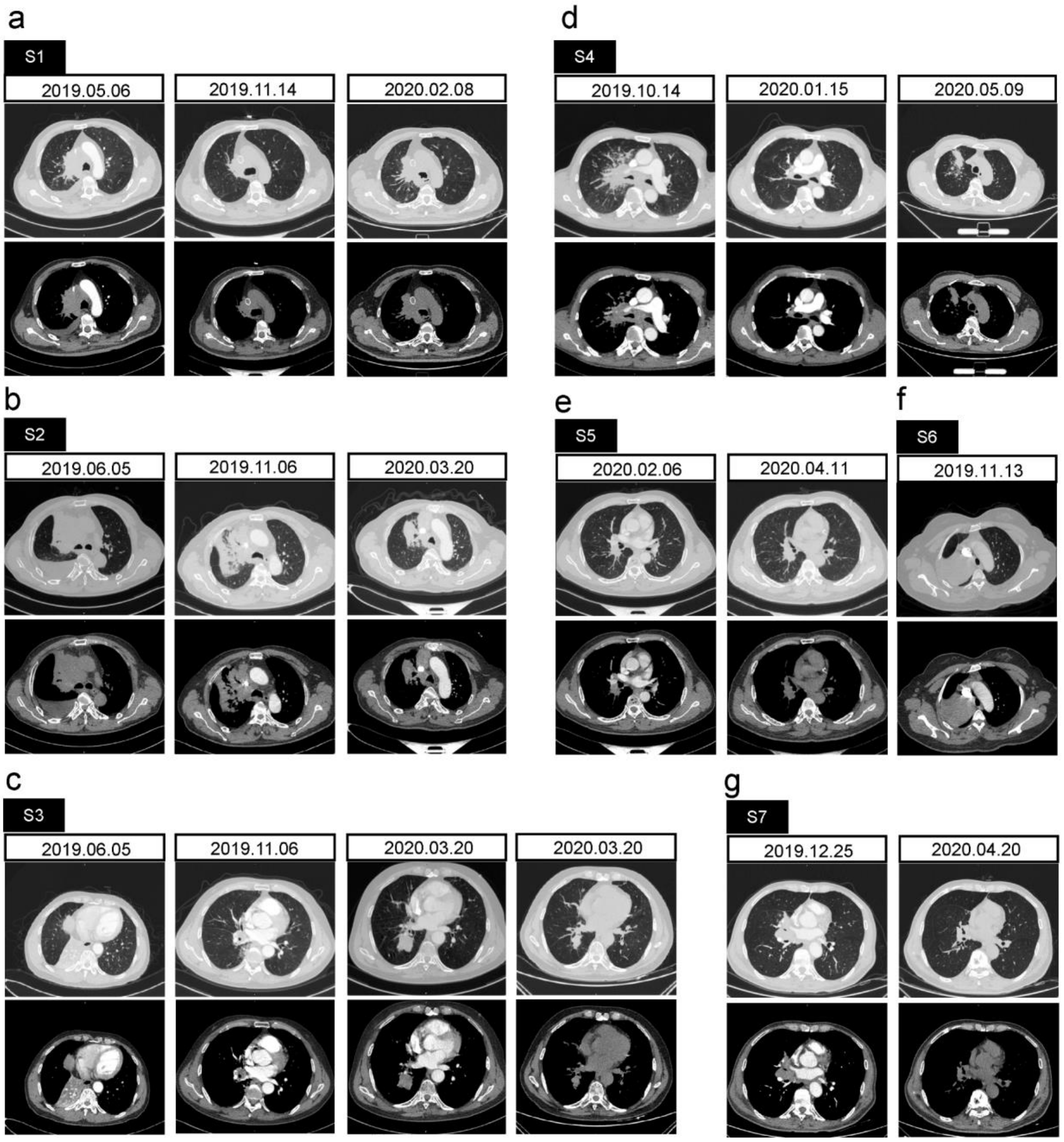

Figure S1. CT scans performed on patients at diagnosis and during the course of treatment. **a.** Patient SCLC1 (S1). **b.** Patient SCLC2 (S2). **c.** Patient SCLC3 (S3). **d.** Patient SCLC4 (S4). **e.** Patient SCLC5 (S5). **f.** Patient SCLC6 (S6). **g.** Patient SCLC7 (S7).

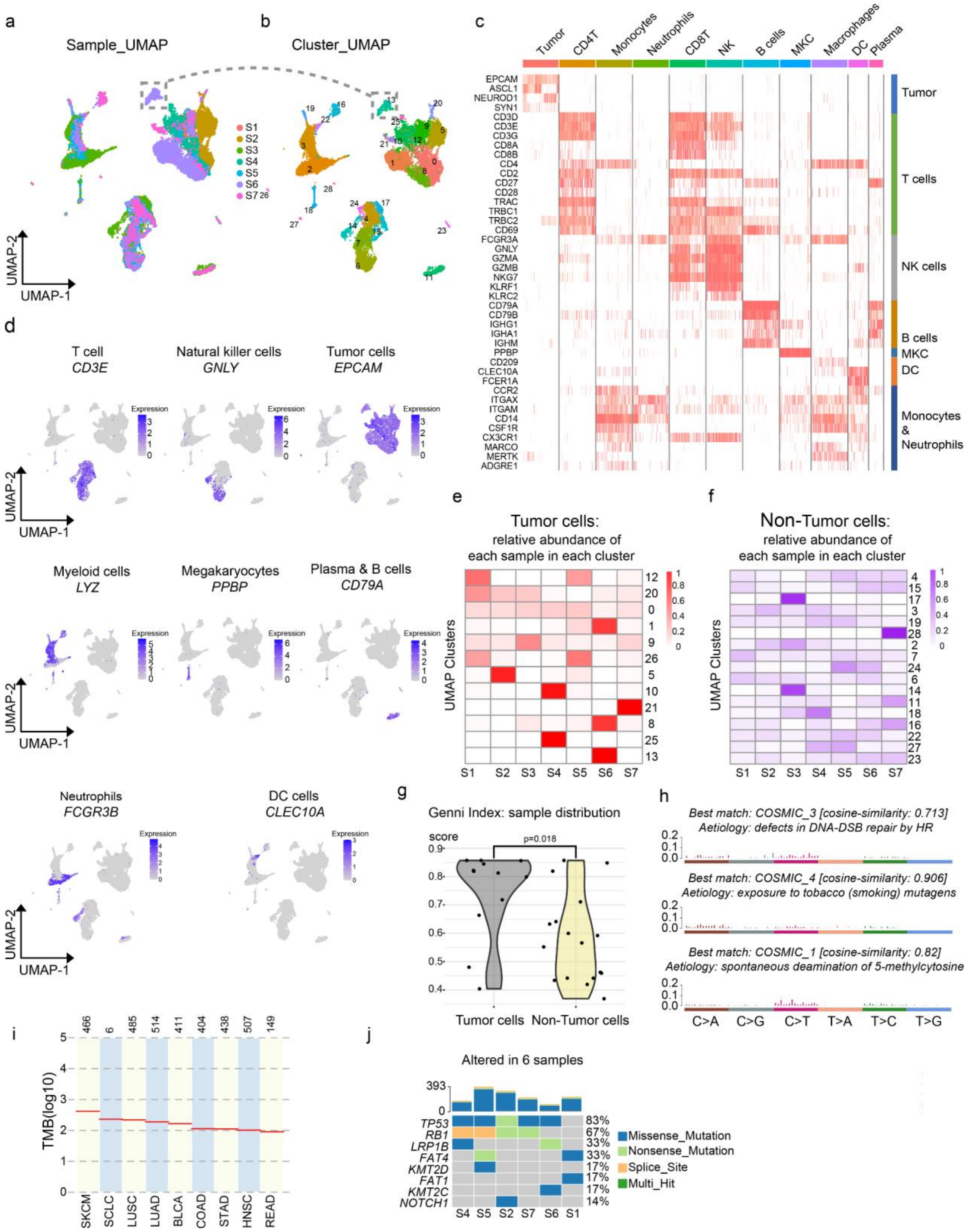

Figure S2. Malignant cells exhibited high interpatient heterogeneity in different SCLC samples. **a, b.** UMAP plot showing distribution of different cells in each patient (**a**) and visualization of different cells reclustered into 29 clusters (**b**). **c.** Heatmap showing signature gene expression in malignant cells, CD4+ T cells, monocytes, neutrophils, CD8+ T cells, NK cells, B cells, MKCs, macrophages, DCs and plasma cells. **d**. UMAP plots showing marker gene expression in T cells, NK cells, malignant cells, myeloid cells, megakaryocytes, plasma and B cells, neutrophils and DC. **e**, **f.**

Heatmap showing the relative abundances of malignant cells (**e**) and non-malignant cells (**f**) in each cluster of each patient. **g.** Gini index of malignant cells is higher than non-malignant cells (p = 0.018). **h.** Mutational signature analysis showing the mutational processes related to DNA DSB repair deficiency (cosine similarity: 0.713), tobacco mutagen exposure (cosine similarity: 0.906) and spontaneous deamination of 5-methylcytosine (cosine similarity: 0.82) in the malignant cells. **i**. TMB in the SCLC samples and other tumors, such as skin cutaneous melanoma (SKCM), lung squamous cell carcinoma (LUSC), lung adenocarcinoma (LUAD), bladder urothelial carcinoma (BLCA), colon adenocarcinoma (COAD), stomach adenocarcinoma (STAD), head and neck squamous cell carcinoma (HNSC), and rectum adenocarcinoma (READ). **j.** WES identified the top mutated genes in the 6 SCLC samples.

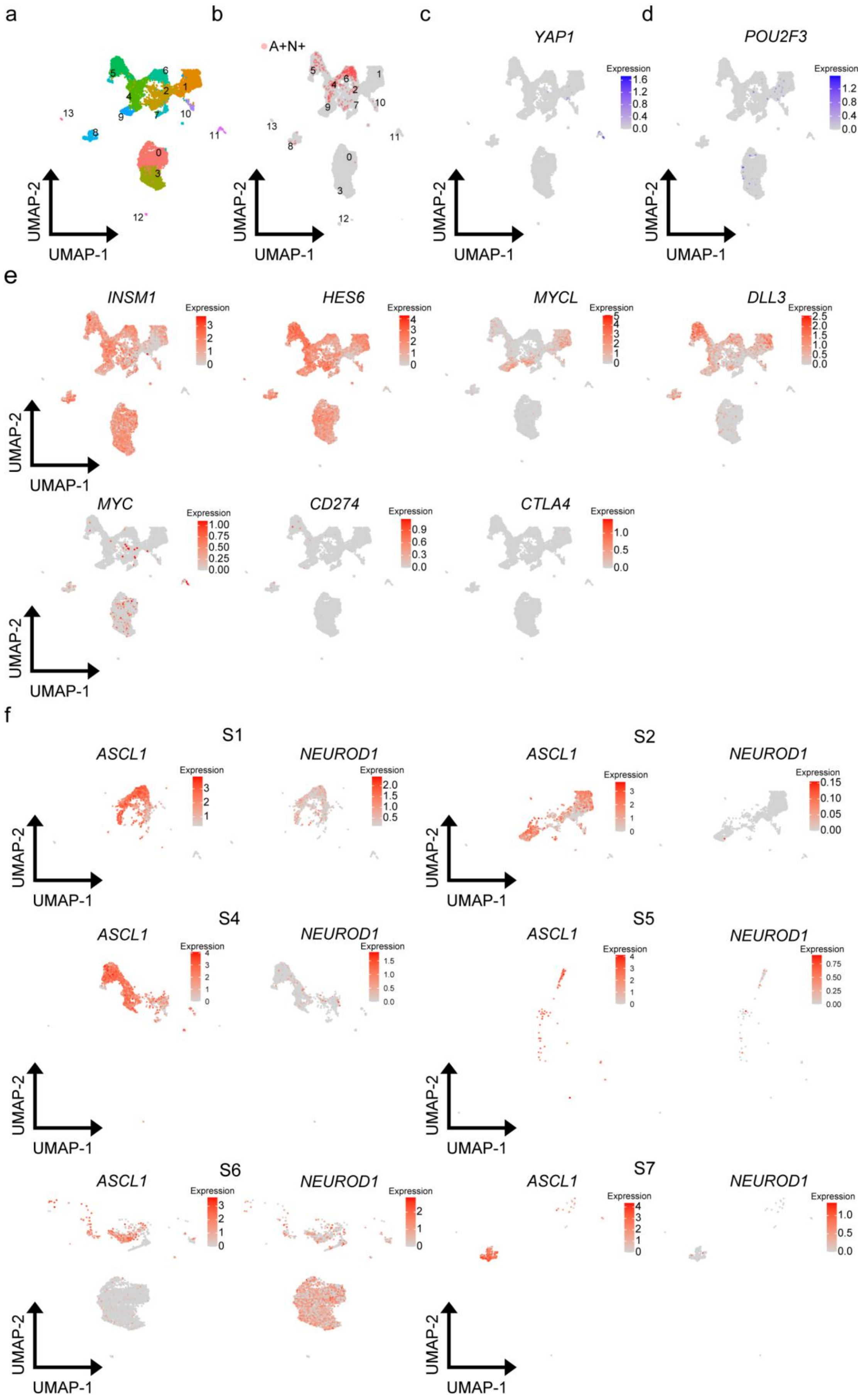

a
b
c
d
A+N+
YAP1
POU2F3
Expression
UMAP-2
UMAP-1
e
INSM1
HES6
MYCL
DLL3
MYC
CD274
CTLA4
f
S1
S2
S4
S5
S6
S7
ASCL1
NEUROD1

Figure S3. Expression of NE and immune markers in all malignant cells profiled by scRNA-seq. **a.** Distribution of different subclusters of malignant cells extracted from all cells by UMAP. **b.** Visualization of malignant cells with high expression of both *ASCL1* and *NEUROD1* (red dot). **c, d.** UMAP plots showing the gene expression of YAP1 and POU2F3 in malignant cells. **e.** UMAP plots showing the expression of *INSM1*, *HES6*, *MYCL*, *DLL3*, *MYC*, *CD274* (PD-L1) and *CTAL4* in the malignant cell. **f.** UMAP plots showing the expression of *ASCL1* and *NEUROD1* in the malignant cell of each sample.

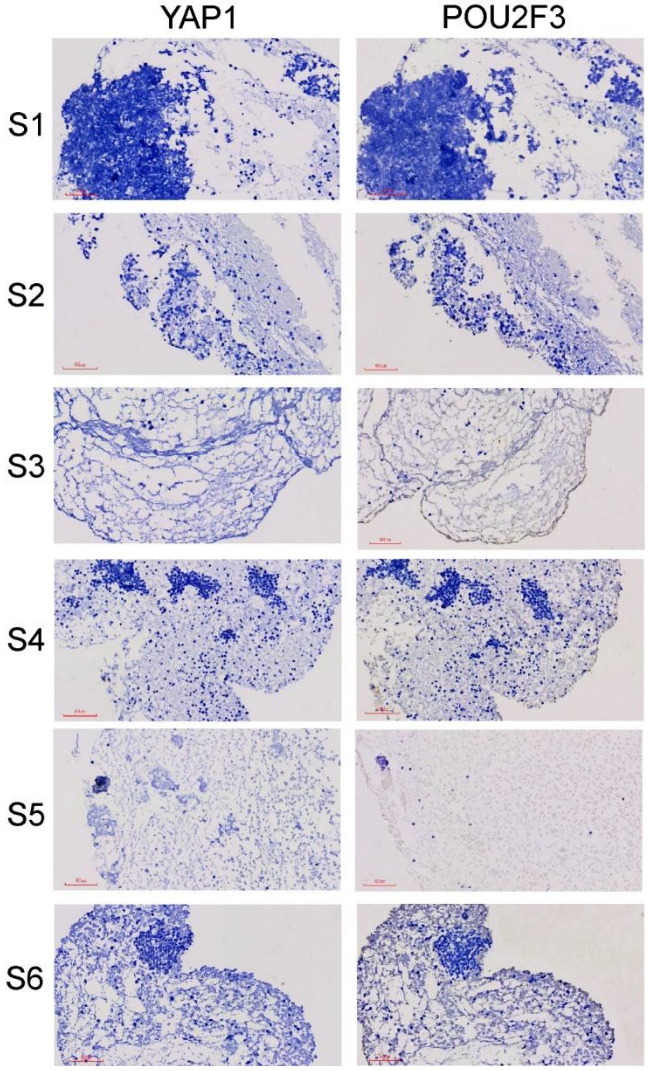

Figure S4. Expression of YAP1 and POU2F3 in different patients, as assessed by IHC. S1, S2, S3, S4, S5 and S6 were negative for YAP1 and POU2F3 expression.

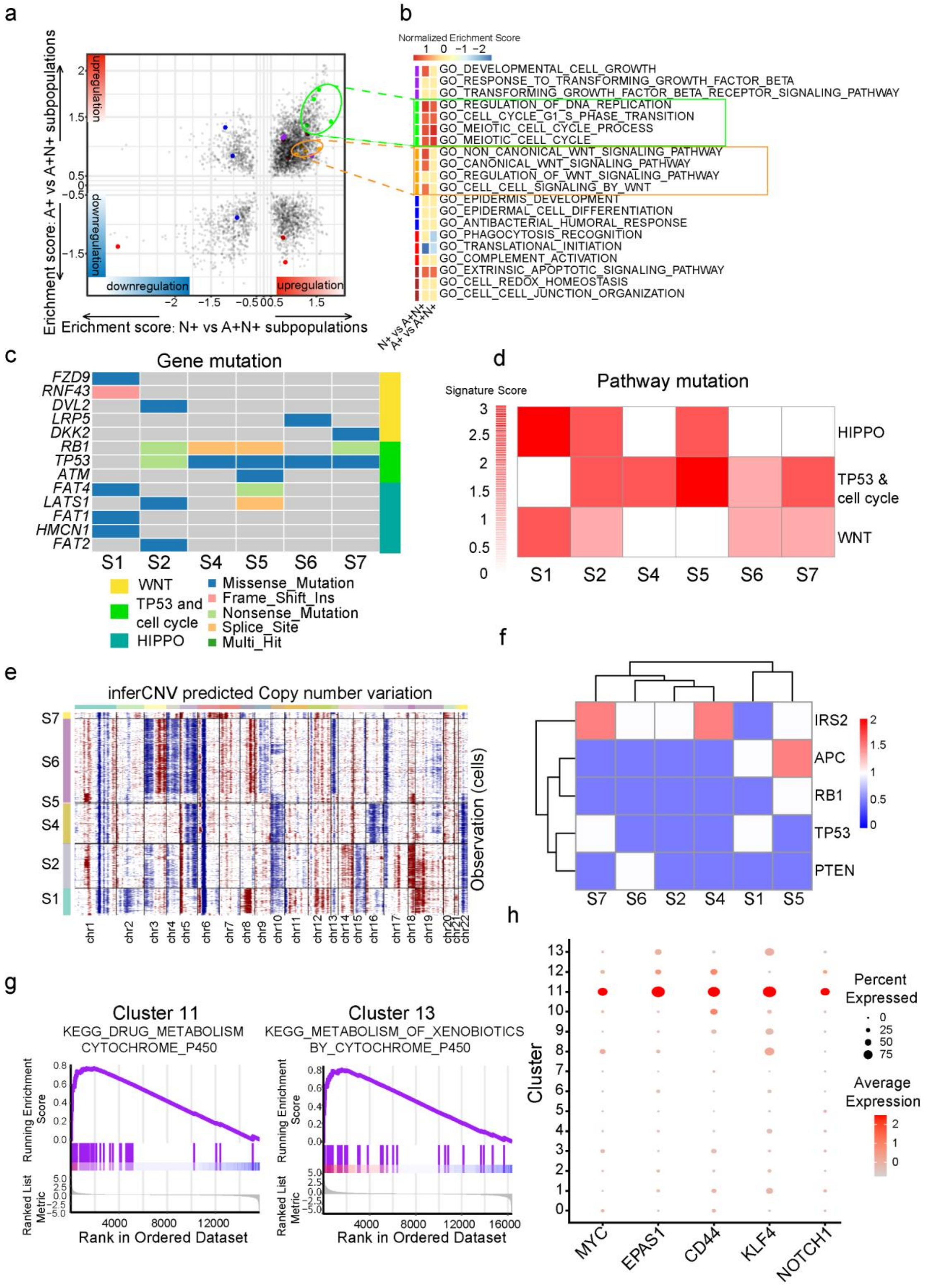

Figure S5. Mutation analysis and copy number analysis. **a**. Scatter plot shows the GSEA normalized enrichment scores (NES) of Gene Ontology (GO) pathways in

either A+ or N + status in comparison to A+N+ status. Positive scores (NES > 0) represent the pathways that are up-regulated in N+ subpopulation (x-axis) or in A+ subpopulation (y-axis) versus A+N+ subpopulation. For instance, green and orange dots represent the pathways that are up-regulated in either A+ or N+ subpopulation compared to A+N+ subpopulation. **b**. The top GO pathways in (**a**) highlighted with different colors. **c**. The mutation frequency of genes in the WNT, TP53 and cell cycle, and Hippo pathways in each patient. **d**. The top mutated genes involved in the canonical WNT, TP53 and cell cycle, and Hippo pathways in each patient. **e.** Heatmap showing the inferred copy number variations (by inferCNV) in each patient. **f.** The CNVs with high frequency in different patients. **g**. GSEA indicated that cluster 11 was positively associated with the drug metabolism-cytochrome p450 pathway and that cluster 13 was positively associated with the metabolism of xenobiotics by cytochrome p450 pathway. **h**. Expression of stemness marker genes in cluster 11.

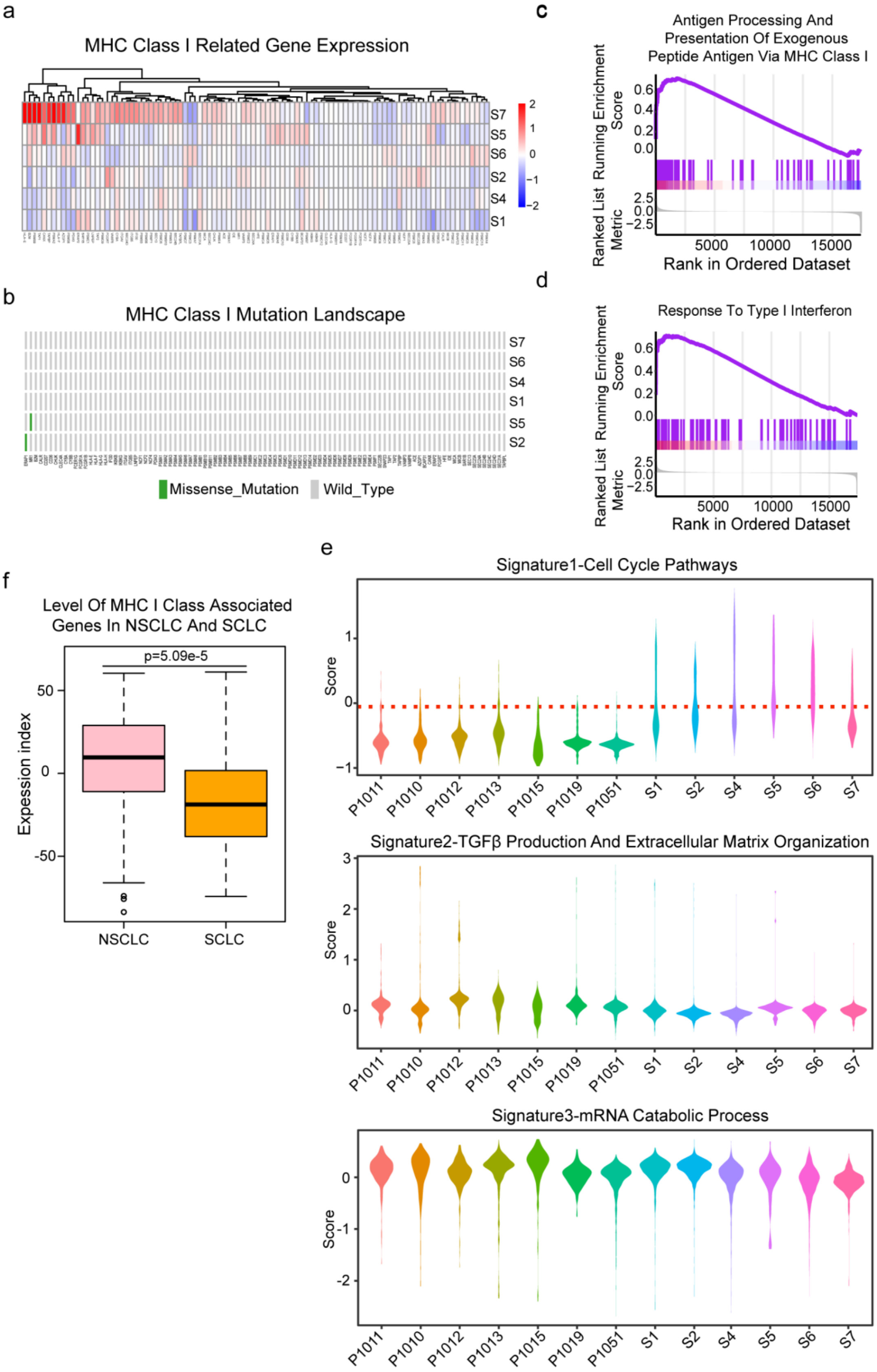

Figure S6. The relation between MHC class I related genes and immune infiltration. **a**. Heatmap showing the expression of genes related to MHC class I molecules in each

patient. **b**. Mutations of in MHC class I related genes were rare in each patient. **c**. GSEA showing that antigen processing and presentation of exogenous peptide antigens via MHC class I related genes were less activated in malignant cells of the patients with lower immune infiltration (S1, S2, S4, S6) than those with higher immune infiltration (S3, S5, S7). **d**. GSEA showing that patients with high immune infiltration was responsive to Type I interferon. **e.** The differences of signature1 associated with cell cycle pathways, signature2 correlated with TGFβ production and extracellular matrix organization, signature3 mainly related with mRNA catabolic process between SCLC and NSCLC were shown via violin plot. **f**. The expression of MHC I class related genes in SCLC cell lines were lower than that of NSCLC cell lines in CCLE database.

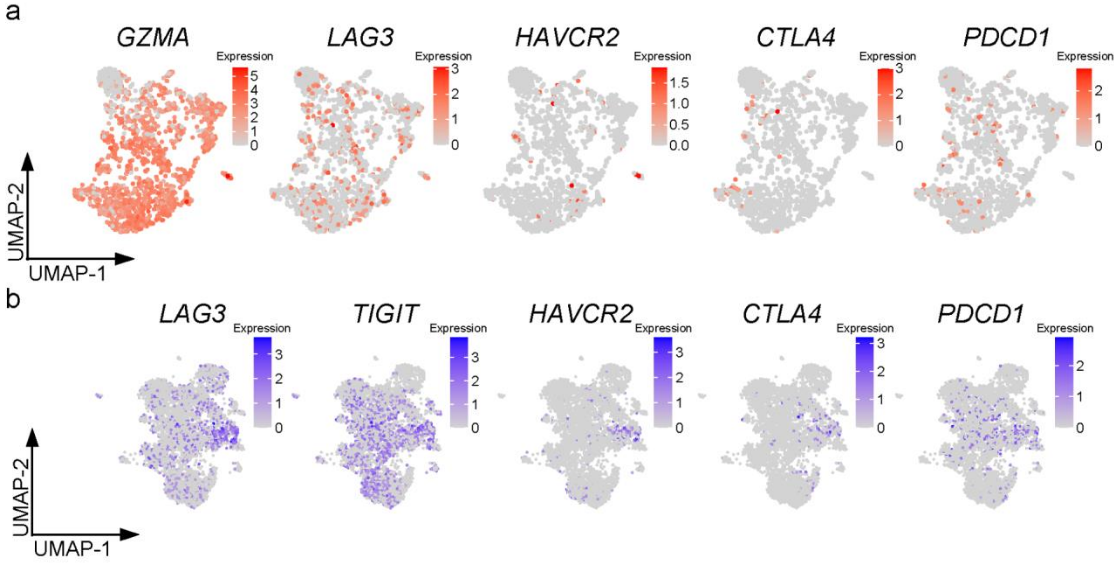

Figure S7. Exhausted CD8+ T cells are not abundant in SCLC patients. **a**. UMAP plots showing high expression of *GZMA* and low expression of known exhaustion markers, including *LAG3*, *HAVCR2*, *CTLA4*, and *PDCD1*, in CD8+ T cells of SCLC. **b**. UMAP plots showing the expression of *LAG3, TIGIT, HAVCR2*, *CTLA4*, and *PDCD1* in CD8+T cells of SCLC and NSCLC.

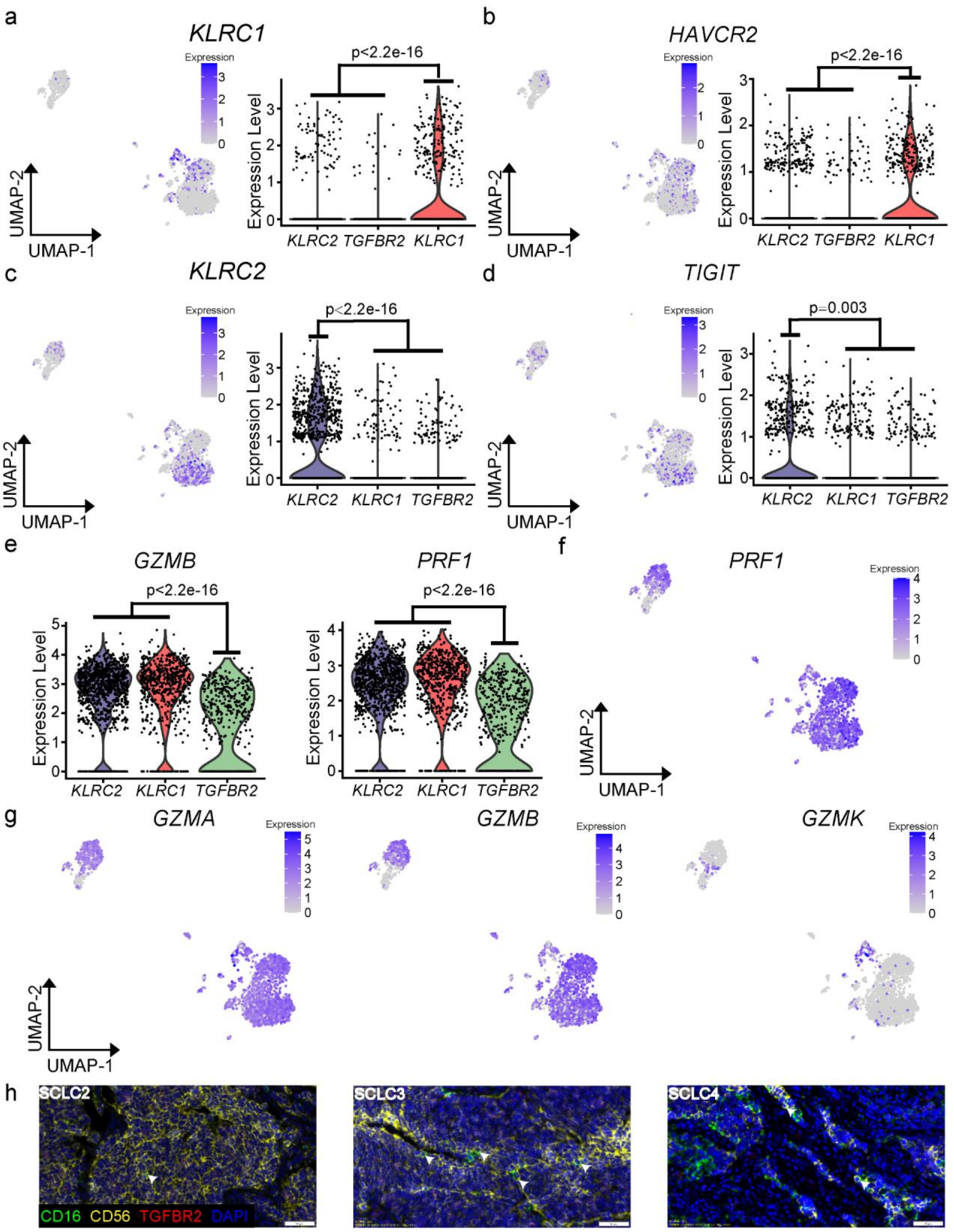

Figure S8. NK cells have impaired cytotoxicity. **a**. UMAP plots showing the KLRC1+ clusters, respectively (left panels); violin plot revealing the higher expression of *KLRC1* (p<2.2e-16) (right panels). **b**. UMAP plot showing the expression of the exhaustion marker HAVCR2 (left panel); scatter plot showing higher expression of *HAVCR2* in the KLRC1+ cluster (right panel, p<2.2e-16) of NK cells. **c**. UMAP plots showing the KLRC2+ clusters, respectively (left panels); violin plot revealing the

higher expression of *KLRC2* (p<2.2e-16) (right panels) in KLRC2+ clusters. **d**. UMAP plot showing the expression of the exhausted marker TIGIT (left panel); scatter plot showing the higher expression of *TIGIT* in the KLRC2+ cluster (right panel, p=0.003) of NK cells. **e**. Lower expression of *GZMB* (p<2.2e-16) and *PRF1* (p<2.2e-16) in TGFBR2+ NK cells. **f**, **g**. UMAP showing the expression of *GZMB*, *GZMA*, *PRF1*, and *GZMK* in all the NK cells from SCLC, NSCLC and healthy controls. **h**. Arrow heads point in representative multiplex immunohistochemistry images showing TGFBR2- NK cells (CD16+ (green) and CD56+ (yellow)) in SCLC.
