# Supplementary material for: Single‐Cell Transcriptome Identifies Drug‐Resistance Signature and Immunosuppressive Microenvironment in Metastatic Small Cell Lung Cancer
Source: Adv Genet (Hoboken). 2022 Mar 4;3(2):2100060. doi: 10.1002/ggn2.202100060 (PMC9744506; doi:10.1002/ggn2.202100060)
Supplement: Supplementary file 2 — Supplemental Table 1 [file GGN2-3-2100060-s004.pdf]

ADVANCED GENETICS
Open Access

Supporting Information

for *Advanced Genetics*, DOI 10.1002/ggn2.202100060

Single-Cell Transcriptome Identifies Drug-Resistance Signature and Immunosuppressive Microenvironment in Metastatic Small Cell Lung Cancer

Jing Zhang, Haiping Zhang, Lele Zhang, Dianke Li, Mengfan Qi, Liping Zhang, Huansha Yu, Di Wang, Gening Jiang, Xujun Wang, Xianmin Zhu* and Peng Zhang**

Supplementary table1. characteristics of SCLC patients

ID	Age	Sex	Smoking history	Stage	EBUS Location	Chemotherapy	Pathology	Ki67	CD56	TTF1	CK	P40	Syn	Chr-a
S1	68	M	Yes	cT4N3M1b(IVA)	4R	resistant	SCLC	-	+	+	+	-	+	+
S2	67	M	Yes	cT4N3Mx (IIIC)	4R,7	resistant	SCLC	+	-	+	N/A	-	+	-
S3	67	M	Yes	cT4N2M1a(IVA)	7	sensitive	SCLC	+	N/A	-	-	-	-	-
S4	70	M	Yes	cT4N2M0(IIIB)	7	sensitive	SCLC	-	-	+	+	-	+	-
S5	53	M	Yes	cT2aN2M0(IIIA)	11R	sensitive	SCLC	+	+	+	+	+	+	-
S6	46	F	No	cT4N2M0 (IIIB)	4R	Unknown	SCLC	+	+	+	-	-	-	-
S7	66	M	Yes	cT4N3M1C(IVB)	7	sensitive	SCLC	+	+	+	+	-	+	-
